# Morphological Characterization and Gene Expression Profiling during Bud Development in a Tropical Perennial, *Litchi chinensis* Sonn.

**DOI:** 10.3389/fpls.2016.01517

**Published:** 2016-10-26

**Authors:** Huifen Zhang, Hua Li, Biao Lai, Haoqiang Xia, Huicong Wang, Xuming Huang

**Affiliations:** ^1^Guangdong Litchi Engineering Research Center, College of Horticulture, South China Agricultural UniversityGuangzhou, China; ^2^Gene Denovo Biotechnology Co. Ltd.Guangzhou, China

**Keywords:** *Litchi chinensis* Sonn., bud development, dormancy, RNA-seq, transcriptomics, gene profiling, short vegetative proteins

## Abstract

Tropical evergreen perennials undergo recurrent flush growth, and their terminal buds alternate between growth and dormancy. In sharp contrast to the intensive studies on bud development in temperate deciduous trees, there is little information about bud development regulation in tropical trees. In this study, litchi (*Litchi chinensis* Sonn.) was used as a model tropical perennial for morphological characterization and transcriptomic analysis of bud development. Litchi buds are naked with apical meristem embraced by rudimentary leaves, which are brown at dormant stage (Stage I). They swell and turn greenish as buds break (Stage II), and as growth accelerates, the rudimentary leaves elongate and open exposing the inner leaf primodia. With the outgrowth of the needle-like leaflets, bud growth reaches a maximum (Stage III). When leaflets expand, bud growth cease with the abortion of the rudimentary leaves at upper positions (Stage IV). Then buds turn brown and reenter dormant status. Budbreak occurs again when new leaves become hard green. Buds at four stages (Stage I to IV) were collected for respiration measurements and in-depth RNA sequencing. Respiration rate was the lowest at Stage I and highest at Stage II, decreasing toward growth cessation. RNA sequencing obtained over 5 Gb data from each of the bud samples and *de novo* assembly generated a total of 59,999 unigenes, 40,119 of which were annotated. Pair-wise comparison of gene expression between stages, gene profiling across stages, GO/KEGG enrichment analysis, and the expression patterns of 17 major genes highlighted by principal component (PC) analysis displayed significant changes in stress resistance, hormone signal pathways, circadian rhythm, photosynthesis, cell division, carbohydrate metabolism, programmed cell death during bud development, which might be under epigenetic control involving chromatin methylation. The qPCR results of 8 selected unigenes with high PC scores agreed with the RPKM values obtained from RNA-seq. Three Short Vegetative Phase (SVP) genes, namely *LcSVP1, LcSVP2*, and *LcSVP3* displayed different expression patterns, suggesting their differential roles in bud development regulation. The study brought an understanding about biological processes associated with the phase transitions, molecular regulation of bud development, as well as cyclic bud growth as a strategy to survive tropical conditions.

## Introduction

Dormancy, an important phase of bud development is considered as a survival strategy taken by plants to survive seasonal harsh climatic conditions. Winter dormancy is found in temperate deciduous trees for surviving winter freezing (Rohde and Bhalerao, 2007). Plants grown in a Mediterranean climate develop summer dormancy to endure the extreme hot and dry conditions in the summer season (Ofir and Kigel, 2007). Based on the causes, three statuses of dormancy can be distinguished: paradormancy, ecodormancy, and endodormancy (Lang et al., 1987), where growth arrest is imposed by apical dominance, adverse environmental conditions and endogenous state of the bud *per se*, respectively. The development and removal of winter dormancy in bud of deciduous trees involves transitions between dormancy statuses (Arora et al., 2003; Anderson et al., 2010; Díaz-Riquelme et al., 2012). Endodormancy in these plants is the innate dormant state induced by short day photoperiod and/or low temperatures and is released by exposure to chilling temperatures for a period of time (Davis, 2002; Arora et al., 2003; Anderson et al., 2010).

Unlike deciduous temperate trees whose annual cycle consists of growth in spring and summer, growth cessation and bud set in autumn and dormancy in winter, many evergreen subtropical and tropical trees, such as litchi, citrus, mango and longan are characterized by recurrent growth with a number of flush growth cycles occurring in a year (Whiley et al., 1989; Fu et al., 2014). Litchi displays a cyclic growth pattern even in a constant controlled environment, suggesting endogeneity of the rhythm (Hieke et al., 2002; O'Hare and Turnbull, 2004). Hence, the terminal buds of the like species alternate between growth and rest phases at a higher frequency than deciduous trees (Fu et al., 2014). O'Hare and Turnbull (2004) and Wilkie et al. (2008) used “dormancy” to term the growth rest between flush growth. In litchi, cambium maintains active even during bud rest period, when shoot thickening continues while shoot elongation has stopped (Fu et al., 2014). Therefore, the dormancy during the growth check period in litchi is highly localized and limited to the terminal meristem in buds. Obviously, this type of dormancy is evolved not entirely for coping with harsh environmental conditions, although harsh conditions do induce growth rest or suppress flushing in these species (Menzel and Simpson, 1994; O'Hare and Turnbull, 2004). The dormancy in litchi bud is neither paradormancy, as it occurs in the terminal buds, nor ecodormancy, as it occurs even in constant conditions favorable for growth (Hieke et al., 2002). Therefore, if refered to the classification of dormancy defined by Lang et al. (1987), the dormancy of litchi bud may fall in the category of endodormancy. However, the endodormancy in litchi is not induced or released by environmental cues but a purely endogenous process, distinguishing it from the endodormancy in temperate trees. The unique cyclic flushing pattern of the evergreen tropical trees in a constant growth-favorable environment raises a profound question: why is it required?

Mechanisms of bud dormancy development and release are intriguing subjects for research. In sharp contrast to the intensive studies conducted in and the deep understanding about the regulation of bud dormancy in temperate tree species, there is much less information about the regulation of bud dormancy in tropical evergreens. However, the understandings of dormancy in temperate species provide convenient reference for studying developmental dormancy in tropical evergreens.

Dormancy development in some temperate trees is initiated by sensing short photoperiod, which induces apical growth cessation, bud set, and the subsequent chain events leading to dormancy and cold tolerance (Olsen, 2010). Phytochromes, the sensor of photoperiod, have been found to regulate dormancy development. Over-expression of phytochrome A (PHYA) gene led to delayed development of dormancy in *Populus* (Olsen et al., 1997), while reduction of its expression advanced growth cessation and thus dormancy in aspen (Arora et al., 2003). Short photoperiod down-regulates active GA while up-regulates ABA, which is related to the cessation of apical growth and bud set (Olsen, 2003). In poplars, dormancy entrance involves reprograming of transcription and metabolism toward the synthesis of protecting and cold acclimation-related proteins such as dehydrins, heat-shock proteins (HSP) and late embyogenesis abundant proteins (LEA) (Ueno et al., 2013), which is primarily orchestrated by abscisic acid (ABA) and ethylene (Ruttink et al., 2007; Horvath et al., 2008). These hormones participate in bud set and dormancy entrance (Ruonala et al., 2006). Erez et al. (1998) reported that induction and development of bud dormancy in peach was associated with the loss of water activity with the conversion of free water in bud into bound water, while dormancy release was accompanied by increase in free water. Opposite to free water, dehydrin expression levels in bud of Norway spruce increased with induction of dormancy and decreased with bud burst (Yakovlev et al., 2008). Recent studies have shown that sugars may serve as important signals for dormancy entrance and maintenance (Anderson et al., 2005, 2010).

Dormancy release of deciduous trees is naturally induced by chilling temperatures. It can also be achieved by application of dormancy-breaking reagents such as hydrogen cyanamide (HC) and stresses such as high temperatures, desiccation, and anoxia (Lavee and May, 1997; Halaly et al., 2008; Ophir et al., 2009). In grape, Ophir et al. (2009) suggested that dormancy release induced by HC and heat shock involves in down-regulation of tricarboxylic acid cycle (TCA cycle) and ATP synthesis and up-regulation of glycolysis, anaerobic respiration and oxidative stress. And temporary oxidative stress and respiration stress (anoxia) participate in the mechanisms of endodormancy removal (Halaly et al., 2008). Dormancy release is accompanied by a reduction in ABA (Horvath et al., 2008) and up-regulation of mechanisms that reduces sugars (Anderson et al., 2010).

A group of dormancy associated MADS-box (DAM) genes responsible for endodormancy have been identified in peach, and they also regulate growth cessation and terminal bud formation (Bielenberg et al., 2004, 2008). Similar genes have been reported in other deciduous trees such as raspberry (Mazzitelli et al., 2007), apricot (Sasaki et al., 2011) and pear (Ubi et al., 2010). DAM genes are related to the *SHORT VEGETATIVE PHASE* (*SVP*) genes found in Arabidopsis (Bielenberg et al., 2004). They are induced by short day and suppress the flowering and growth maintenance genes such as *FLOWERING LOCUS T* (*FT*) and FT-like *CENTRORADIALIS-LIKE 1* (*CENL1*) (Lee et al., 2007; Horvath et al., 2008). Therefore, DAM genes control flowering as well as dormancy/growth in deciduous trees.

Although, flowering of many tropical fruit crops such as litchi, longan, mango, and avocado is induced by chilling temperatures, growth status of flush exerts critical effect on responsiveness to chilling (Wilkie et al., 2008; Olsen, 2010). In litchi and mango, breaking buds are most inductive to flowering by chilling, while dormant and intensively growing buds lose the competence to chilling, leaving only a very small part of the flush growth cycle for floral induction (Batten and McConchie, 1995; Olsen, 2010). Hence, manipulation of flush growth cycle in such tropical fruit trees is critical for flowering and thus for a crop. In this study, we used in-depth RNA sequencing technique to analyze the transcriptomic changes during bud development so as to bring an understanding of the molecular regulation of bud development, especially the entry and release of developmental dormancy in litchi, a model tropical perennial tree, as well as an understanding of the biological significance of its cyclic growth pattern.

## Methods

### Materials and sample collection

**Figure 1:**
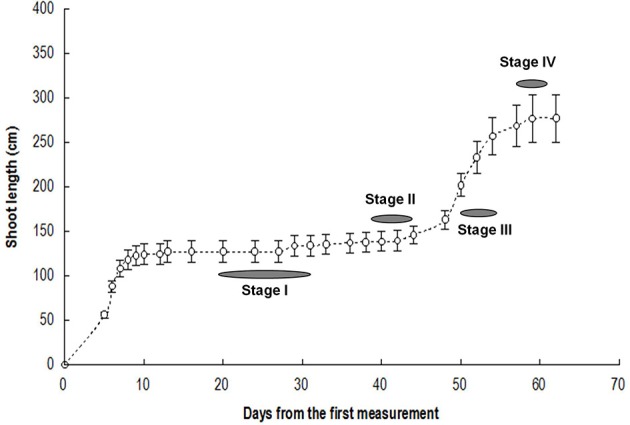
**Flush growth and stages for bud sampling**. Vertical bars represent standard errors of means (*n* = 3).

**Figure 2:**
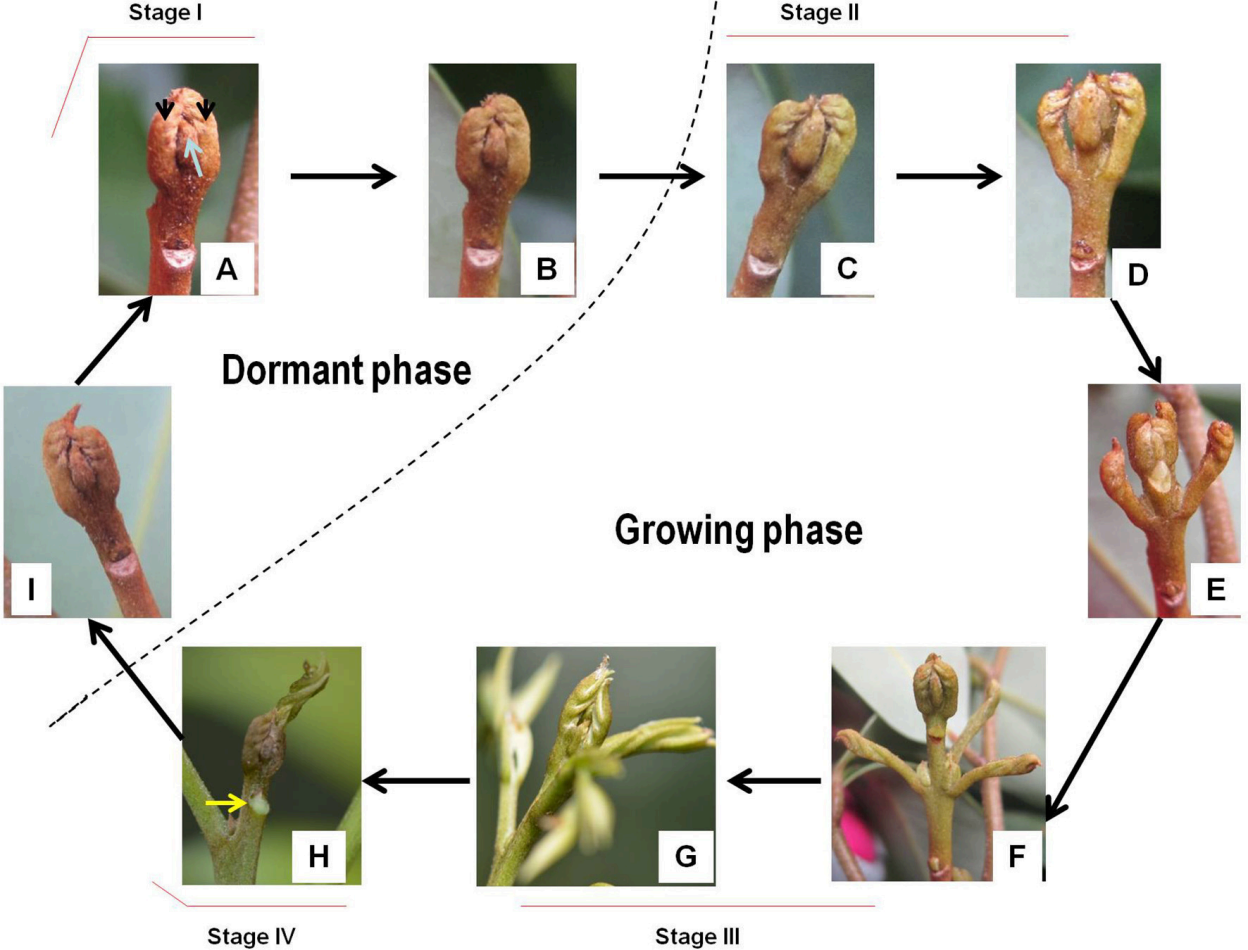
**Morphological changes of litchi bud during the development cycle (A–I)**. Dashed line separates the dormant phase and the growing phase. Arrows in subfigure **(A)** indicate rudimentary leaves or leaf primodia. The arrow in subfigure **(H)** denotes a freshly-formed leaf scar from an abscised leaf at the upper position of the new flush.

Sixteen-year-old trees of *Litchi chinensis* Sonn. cv. Feizixiao growing in the experimental orchard of South China Agricultural University, Guangzhou, China, were used for this study. The trees were slightly pruned leaving about two thirds of the leaves in the early July after harvest to encourage flush growth. When new growth occurred, 10 shoots each from three trees (*n* = 3) were tagged and their lengths traced until a second flush growth has ended in late September. Morphological changes of bud during flush growth cycle were observed and photographed regularly. Terminal buds about 2 mm in length were collected at four different stages shown in Figure 1. Stage I was dormant stage, when buds were brown and the top leaves were fully expanded yet still soft green; Stage II corresponded to bud break stage, when the top leaves were hard green and buds became greenish and slightly swelled; Stage III, the rapid elongation stage, when bud were in active growth sending out new compound leaves with folded leaflets that were needle-like in shape; and Stage IV, the growth cessation stage during leaf expansion, when elongation growth ceased with weak growth or abortion of the upper rudimentary leaves. Bud morphology at the four sampling stages is shown in Figure 2. The bud samples collected were used to measure respiration or immediately put into liquid nitrogen and stored in a freezer at −80°C.

### Measurement of respiration

Oxygen consumption rates of buds freshly harvested at the above mentioned stages were measured using an Oxygraph liquid phase oxygen measurement system (Hansatech, England) under 30°C. The measurement was conducted with 5 replicates (buds from 5 different trees) each consisting of 5 buds from the same tree.

### RNA extraction, cDNA synthesis and sequencing

Sixty frozen buds from each stage were pooled for total RNA extraction, which was carried out using a Quick RNA Isolation Kit (Huayueyang, China) according to the manufacturer's instructions. During the extraction, DNase I (Takara, Japan) was added to remove genomic DNA and total RNA was purified using RNase-free columns (Huayueyang, China). The integrity and quality of RNA extracted was checked by agarose gel electrophoresis and the BioPhotometer Plus photometer (Eppendorf, Germany). The purified total RNA samples were sent to Guangzhou Gene Denovo Biotechnology Co. Ltd, where the samples were processed according to the procedure shown in Figure S1A and cDNA library was separately constructed for each bud stage from the corresponding total RNA samples before sequenced with Illumina HiSeq™ 2000 using the paired-end technology (PE100) by Gene Denovo Co. (Guangzhou, China, http://www.genedenovo.com).

### *De novo* assembly and annotation

*De novo* assembly, unigene annotation, and GO and pathway enrichment analyses were conducted following the procedure shown in Figures S1B,C. After removing the raw reads containing adapter, reads with more than 5% unknown nucleotides, and low quality reads with the percentage of low *Q*-value (≤ 10) base higher than 20%, clean reads were *de novo* assembled by the Trinity program into contigs (Grabherr et al., 2011), which were further processed with TIGR Gene Indices clustering tools (TGICL) that effectively removes redundancy (Pertea et al., 2003). The unigenes thus generated were annotated using BLASTx program (http://www.ncbi.nlm.nih.gov/BLAST/ webcite) with an *E*-value threshold of 1e−5 to NCBI nr database (http://www.ncbi.nlm.nih.gov webcite), the Swiss-Prot protein database (http://www.expasy.ch/sprot webcite), the KEGG database (http://www.genome.jp/kegg webcite), and the COG database (http://www.ncbi.nlm.nih.gov/COG webcite). KEGG pathway annotation was done using BLASTx program (http://www.ncbi.nlm.nih.gov/BLAST/ webcite) against the KEGG database.

### Differentially expressed genes (DEG) between stages and function enrichment

Clean reads were mapped to reference sequence by the SOAPaligner/soap2, a tool designed for short sequences alignment. Reads uniquely mapped to a unigene were used to calculate the expression level, which was expressed as reads per kilobase of exon region per million mappable reads (RPKM). After the expression level of each gene was calculated, differential expression analysis was conducted using edgeR (Robinson et al., 2010). The false discovery rate was used to determine the threshold of the *p*-value in multiple tests, and for the analysis, a threshold of the FDR ≤ 0.05 and an absolute value of log2Ratio ≥ 1 were used to judge the significance of the gene expression differences. The up- and down-regulated DEGs between stages were separately subjected to GO and KEGG pathway enrichment analyses according to a method similar to that described by Zhang et al. (2013). Top 10 most significantly enriched biological processes were highlighted.

### Gene expression profiling and KEGG pathway enrichment

Gene expression trends from Stage I to Stage IV were analyzed and clustered using the software of Short Time-series Expression Miner (STEM) (Ernst and Bar-Joseph, 2006). Genes were clustered into 26 expression profiles. Those profiles with *P* < 0.01 were separately subjected to KEGG pathway enrichment, and top five most significant pathways were focused.

### Principal component (PC) analysis

RPKM data from the four samples were imported to Robin software suite (http://www.r-project.org/) to perform data normalization using the RMA method. Principal component analysis was performed using fast prcomp functions (Molecular Devices, LLC, CA, US). The score matrix was used to select probe-sets that best fit the first principal component (PC1) and PC2. Major genes with high PC1 or PC2 scores (> = 0.1) were highlighted and clustered based on their expression patterns.

### Real-time PCR of selected genes

Eight genes including 2 annotated as vegetative storage proteins (VSP), 2 seed protein-like isoform X1 (SPL X1), 1 germin-like proteins (GL), 1 proline-rich protein precursor (PRPP), 1 metallothionein (MT), and 1 late embryogenesis abundant protein (LEA) were selected for q-PCR analysis. Based on the known sequences, primers for quantitative real-time PCR (q-PCR) were designed using Primer Premier 5.0 software (Premier, Canada) and synthesized by Sangon Biotech (Shanghai) Co., Ltd. Sequences of primer pairs were shown in Table S1. The litchi *actin* (GenBank accession number: HQ588865.1) was selected as reference (Zhang H. N. et al., 2014). qPCR was performed with three independent biological and two technical replicates on a Bio-Rad iQ5 Optical System Real Time PCR System (Bio-Rad, USA) using a SYBR Green based PCR assay. Each reaction mixture was 20 μL containing 6 μL of diluted first-strand cDNAs and 250 nM of each primer, and 10 μL SYBR Green PCR Master Mix (TaKaRa, Japan). The qPCRs were run as follows: 50°C for 2 min, 95°C for 10 min, followed by 40 cycles of 95°C for 30 s, 56°C for 30 s, and 72°C for 30 s in 96-well optical reaction plates (Bio-rad, USA). Expression levels of the tested genes were determined by CT values and calculated by 2^−ΔΔCt^ (Livak and Schmittgen, 2001). The analyses were conducted with 3 biological replicates, i.e., buds sampled from 3 trees (*n* = 3).

## Results

### Morphological characterization of bud development

Flush growth of litchi is initiated by terminal bud break. Different from the buds in deciduous trees, which are well protected by bud scales or cataphylls, litchi buds are naked and composed of the apex meristem (growing point), leaf primodia or rudimentary leaves (pinnate) and axially bud primodia. Before bud break, the dormant buds had brown colored rudimentary leaves that tightly embraced each other and the growing point within (Figure 2A). Prior to break, the rudimentary leaves slightly turned green (Figure 2B). As bud break occurred, the rudimentary leaves swelled and gradually opened exposing the inner leaf primodia and axillary bud primodia, and greening of the rudimentary leaves continued (Figures 2C–E). The rudimentary leaves elongated after bud break, and the bud apex continued to grow out green and elongating rudimentary pinnate leaves (Figure 2F). With the outgrowth of the needle-like leaflets, the new flush elongated most rapidly with intensive outgrowth of new leaves from the terminal green bud (Figure 2G). As the leaflets expanded, elongation ceased and the upper rudimentary leaves grew weakly and abscised naturally leaving prominent scars (Figure 2H). Growth cessation could also be observed by narrowed internodes in the upper portion of the new shoots. Then the terminal buds (rudimentary leaves) shrank, turned brown, and reentered dormant status (Figure 2I). Bud break for a second flush growth did not occur until the new leaves became hard green.

We selected buds at dormant stage (Stage I), break stage (Stage II), fast growing stage (Stage III) and growth cessation stage (Stage IV) (Figure 1) for respiration measurement and in-depth RNA sequencing.

### Respiration rate of buds at different stages

**Figure 3:**
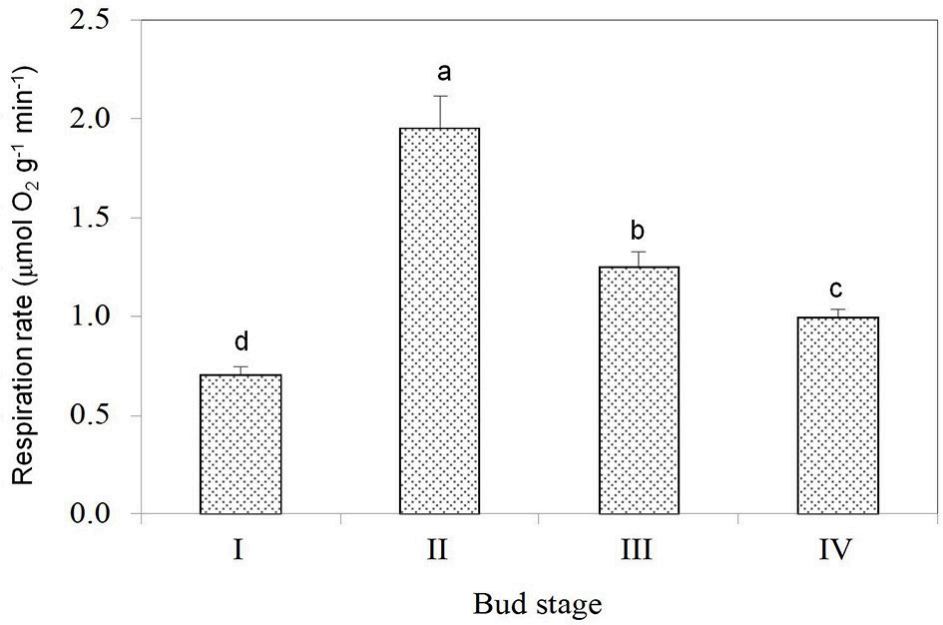
**Respiration rates of buds at different stages**. Vertical bars represent standard errors of means. Different letters above columns indicate significant difference (*P* < 0.05) between stages based on LSD multiple range tests (*n* = 5).

Dormant buds in Stage I had the weakest respiration as reflected by their lowest oxygen consumption rate (Figure 3), suggesting their lowest activity in metabolism. Breaking buds at Stage II had the highest respiration rate, indicating the phase change from dormant state to active growth involved abrupt activation of metabolism processes with high demand for energy. Bud respiration rate was the second highest during the rapid growth stage (Stage III) and lowered as buds entered growth cessation stage.

### The sequencing, assembly, and annotation

The high throughput RNA sequencing obtained over 5 Gb data from each of the bud samples with Q20 all higher than 97.8% and a rate of low quality reads lower than 0.07% (Table 1). The sequencing data are available from the NCBI Short Read Archive (SRA) with an accession number SRP065290 (http://www.ncbi.nlm.nih.gov/sra). Assembly of the reads generated a total of 59,999 unigenes, with an N50 of 770 bp and average length of 558.7 bp (Table 2).

**Table 1:** **RNA Sequencing result**.

**Bud sample**	**Total reads**	**Total nucleotide (nt)**	**Q> = 20 (%)**	**Adapter (%)**	**Low quality (%)**
Stage I	54,558,000	5,455,800,000	97.94	0.04	0.06
Stage II	62,364,000	6,236,400,000	97.88	0.04	0.06
Stage III	55,637,244	5,563,724,400	97.89	0.02	0.06
Stage IV	57,772,766	5,777,276,600	97.86	0.03	0.07

**Table 2:** **Results of *de novo* assembly**.

**Unigene**	**GC%**	**N50**	**Max length**	**Min length**	**Average length**	**Total assembled bases**
59,999	43.31	770	10,827	201	558.7	33,521,309

**Figure 4:**
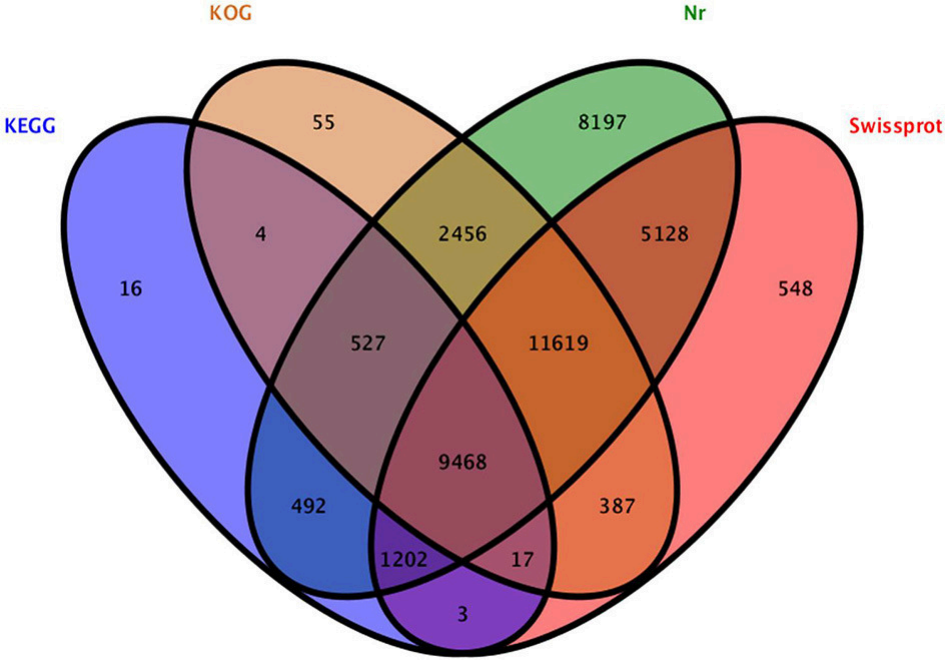
**Venn diagram of unigenes annotated to the four databases**.

A total of 40,119 unigenes could be annotated by BLAST in any of the four databases (nr, Swiss-Prot, KOG, and KEGG), leaving 19,880 (33.1%) unigenes without annotation. A total of 39,089, 28,372, 24,533, and 11,729 unigenes were annotated in nr, Swiss-Prot, KOG, and KEGG databases (Figure 4), respectively, and more than 40% of them displayed high homology (1e < 10^−50^) to known sequences. GO classification and KOG function classification of all the unigenes are shown in Figure S2.

### Differentially expressed genes between stages and GO analysis

**Figure 5:**
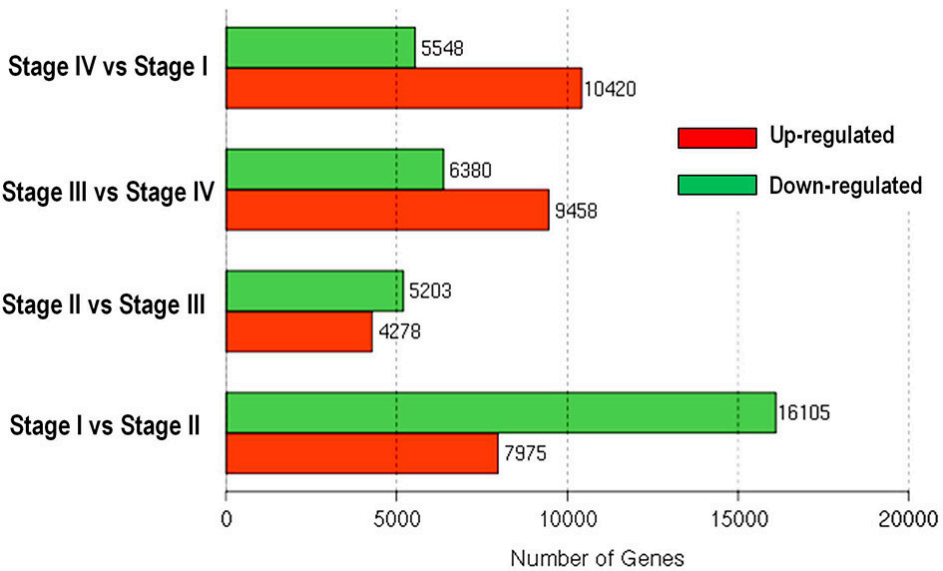
**Numbers of differentially expressed genes involved in phase changes during bud development of litchi**.

Differential expression analyses revealed changes in gene expression during transition between stages (Figure 5). Transition from dormant stage (Stage I) to bud break stage (Stage II) had the greatest number of differentially expressed genes (24,080) with 16,105 down-regulated and 7975 up-regulated genes. Stage II and Stage III had the smallest difference in gene expression, with 5203 down-regulated and 4278 up-regulated during the transition from Stage II to Stage III. From Stage III to Stage IV, 6380 unigenes were down-regulated and 9458 up-regulated. The transition to dormant stage (Stage I) from Stage IV was accompanied by up-regulation of 10,420 and down-regulation of 5548 unigenes.

Pair-wise comparison of differentially expressed genes between successive stages revealed global changes in gene expression during phase transitions of bud development. Ten biological processes most significantly enriched by GO analyses of the up- and down-regulated genes during phase transitions are listed in Tables 3–**6**.

**Table 3:** **Changes in biological processes in buds indentified by GO analysis during Stage I to Stage II transition**.

**Change pattern**	**Gene ontology term**	**Cluster frequency (%)**	***P*-value**	**Corrected *P*-value**
Up-regulated	Histone lysine methylation	3.0	1.78E-27	2.07E-24
	Cell division	3.4	2.86E-27	3.33E-24
	Microtubule-based process	4.4	2.23E-26	2.60E-23
	Peptidyl-lysine methylation	3.1	3.37E-26	3.92E-23
	Macromolecule methylation	4.5	4.87E-25	5.67E-22
	Cytoskeleton-dependent cytokinesis	2.9	1.62E-24	1.89E-21
	Cell cycle process	5.3	1.83E-24	2.13E-21
	Regulation of cell cycle phase transition	1.5	2.20E-24	2.56E-21
	Regulation of mitotic cell cycle phase transition	1.5	2.20E-24	2.56E-21
	Cytokinesis	3.0	1.04E-23	1.21E-20
Down-regulated	Cellular response to acid chemical	1.4	1.27E-06	0.00155
	Signal transduction	7.9	2.71E-06	0.0033
	Signaling	8.0	3.11E-06	0.00378
	Single organism signaling	8.0	3.11E-06	0.00378
	Energy derivation by oxidation of organic compounds	1.0	4.80E-06	0.00584
	Salicylic acid mediated signaling pathway	0.9	5.54E-06	0.00674
	Cellular response to salicylic acid stimulus	0.9	5.54E-06	0.00674
	Regulation of cell death	1.6	6.18E-06	0.00752
	Regulation of programmed cell death	1.6	6.18E-06	0.00752
	Response to salicylic acid	0.9	7.33E-06	0.00892

*Top 10 most significant processes are displayed*.

During the transition from Stage I to Stage II, which involved removal of bud dormancy, the most significantly enriched up-regulated biological processes were involved in epigenetic regulation (e.g., histone lysine methylation, peptidy-lysine methylation, and macromolecule methylation) and cell mitosis/division (e.g., cell division, cell cycle process, and cytokinesis), while the down-regulated processes included cellular response to acid chemical, signal transduction, salicylic acid signaling and response, cell death, and energy derivation by oxidation of organic compounds (Table 3). The results suggest that removal of bud dormancy in litchi involves epigenetic regulation, activation of cell division, reduction in sensitivity to salicylic acid, and deactivation of programmed cell death.

The transition from Stage II to Stage III is a process of growth acceleration. The up-regulated biological processes include mitosis/cell division and nucleic acid metabolism, which agrees with the growth acceleration. The most significant down-regulated processes involved gene expression, purine-containing compound metabolism, ribonucleoside triphosphate metabolism, and tricarboxylic acid metabolism (Table 4). The down-regulation of gene expression agreed with the smallest number of differentially expressed genes between the two stages (Figure 5), while reduced tricarboxylic acid metabolism was in accordance with the reduced respiration rate in Stage III compared with Stage II (Figure 3).

**Table 4:** **Changes in biological processes in buds indentified by GO analysis during Stage II to Stage III transition**.

**Change pattern**	**Gene Ontology term**	**Cluster frequency (%)**	***P*-value**	**Corrected *P*-value**
Up-regulated	Mitotic cell cycle	4.7	4.62E-21	4.30E-18
	Cytoskeleton-dependent cytokinesis	4.0	1.73E-19	1.61E-16
	Mitotic cell cycle process	4.2	2.33E-19	2.16E-16
	Cell division	4.4	5.06E-19	4.70E-16
	Cytokinesis	4.0	1.05E-18	9.81E-16
	Cell cycle process	6.6	1.78E-18	1.66E-15
	DNA metabolic process	12.3	7.87E-18	7.32E-15
	Nucleic acid metabolic process	23.2	9.27E-18	8.62E-15
	Microtubule-based process	5.3	2.77E-17	2.58E-14
	Mitotic cytokinesis	3.7	5.17E-17	4.81E-14
Down-regulated	Gene expression	15.5	9.34E-18	7.18E-15
	Purine ribonucleotide biosynthetic process	2.6	2.35E-14	1.80E-11
	Purine nucleotide biosynthetic process	2.6	8.36E-14	6.43E-11
	Purine ribonucleotide metabolic process	2.8	1.03E-13	7.93E-11
	Citrate metabolic process	2.1	1.71E-13	1.31E-10
	Tricarboxylic acid metabolic process	2.1	1.71E-13	1.31E-10
	Purine-containing compound biosynthetic process	2.6	8.79E-13	6.76E-10
	Ribonucleoside triphosphate metabolic process	1.9	4.07E-12	3.13E-09
	Ribonucleoside triphosphate biosynthetic process	1.9	4.07E-12	3.13E-09
	Ribonucleotide biosynthetic process	2.6	7.56E-12	5.81E-09

*Top 10 most significant processes are displayed*.

A set of biological processes were up-regulated during the transition from Stage III to Stage IV, i.e., from rapid growth to growth cessation. These included cell recognition, glycolipid metabolism, liposaccharide metabolism, sulfate transport, programmed cell death, and response to and signaling of salicylic acid (Table 5). The up-regulation of response to salicylic acid during growth cessation is opposite to its down-regulation during dormancy removal (transition from Stage I to Stage II). Therefore, salicylic acid might play an important role in regulation of bud development cycle in litchi. Among the down-regulated processes during Stage III to Stage IV transition, cell cycle/division and macromolecule methylation/chromosome organization were the most significantly enriched processes (Table 5), which is opposite to the transition from Stage I to Stage II. The down-regulation of cell division/cycle processes agrees well with the reduced growth of bud. The results also indicate that the occurrence of bud growth cessation may be under the epigenetic control.

**Table 5:** **Changes in biological processes in buds indentified by GO analysis during Stage III to Stage IV transition**.

**Change pattern**	**Gene ontology term**	**Cluster frequency (%)**	***P*-value**	**Corrected *P*-value**
Up-regulated	Cell recognition	1.4	2.86E-08	2.90E-05
	Glycolipid metabolic process	1.4	1.22E-07	0.00012
	Liposaccharide metabolic process	1.4	1.22E-07	0.00012
	Sulfate transport	0.5	8.91E-07	0.0009
	Sulfur compound transport	0.5	8.91E-07	0.0009
	Glycolipid biosynthetic process	1.1	2.82E-06	0.00285
	Response to salicylic acid	1.1	5.49E-06	0.00556
	Regulation of cell death	1.8	6.96E-06	0.00704
	Regulation of programmed cell death	1.8	6.96E-06	0.00704
	Salicylic acid mediated signaling pathway	1.1	1.30E-05	0.01324
Down-regulated	Cell cycle process	7.0	2.27E-46	2.61E-43
	Cell cycle	8.6	1.41E-41	1.62E-38
	Macromolecule methylation	5.6	8.59E-40	9.87E-37
	Methylation	5.7	4.82E-39	5.54E-36
	Regulation of DNA metabolic process	3.5	1.18E-38	1.36E-35
	Histone lysine methylation	3.7	3.35E-37	3.85E-34
	Peptidyl-lysine methylation	3.8	1.66E-36	1.90E-33
	Cell division	4.1	4.10E-35	4.71E-32
	Chromosome organization	8.7	5.11E-35	5.87E-32
	Cellular nitrogen compound metabolic process	28.6	8.82E-34	1.01E-30

*Top 10 most significant processes are displayed*.

Following bud growth cessation, the bud entered dormant status, which occurs during the transition from Stage IV to Stage I. The up-regulated biological processes based on GO analysis included tricarboxylic acid metabolism, gene expression, purine-containing compound metabolism, and proton transmembrane transport (Table 6). The increased tricarboxylic metabolisms seemed to be contradictory to the reduced oxygen consumption rate (Figure 3) at Stage I compared with at Stage IV. The lower oxygen consumption rate in dormant bud is a result of lowered operation of oxidative phosphorylation and respiratory electron transport chain. The up-regulated process of gene expression during the transition from Stage IV to Stage I suggests that the entrance to bud dormancy involves massive expression of new genes, agreeing with the results shown in Figure 5. Proton transmembrane transport is associated with uptake and accumulation of solutes in cells. Therefore, entrance of dormancy is accompanied by accumulation of assimilates in buds. The down-regulated biological processes during Stage IV–Stage I transition were mostly related to cell wall biosynthesis, which agrees with the ceased growth with low cell wall synthesis in dormant buds.

**Table 6:** **Changes in biological processes in buds indentified by GO analysis during Stage IV to Stage I transition**.

**Change pattern**	**Gene ontology term**	**Cluster frequency (%)**	***P*-value**	**Corrected *P*-value**
Up-regulated	Citrate metabolic process	2.0	1.24E-21	1.31E-18
	Tricarboxylic acid metabolic process	2.0	1.24E-21	1.31E-18
	Gene expression	13.3	6.17E-14	6.54E-11
	Purine-containing compound metabolic process	2.7	5.21E-13	5.53E-10
	Purine-containing compound biosynthetic process	2.1	1.04E-12	1.11E-09
	Purine nucleotide metabolic process	2.5	1.44E-12	1.53E-09
	Purine ribonucleotide metabolic process	2.2	2.13E-12	2.26E-09
	Hydrogen ion transmembrane transport	1.3	3.31E-12	3.52E-09
	Purine nucleotide biosynthetic process	1.9	1.24E-11	1.32E-08
	Energy coupled proton transmembrane transport, against electrochemical gradient	1.2	9.80E-11	1.04E-07
Down-regulated	Cell wall organization or biogenesis	4.1	4.26E-08	4.02E-05
	Plant-type cell wall biogenesis	0.8	9.22E-08	8.70E-05
	Cell wall biogenesis	1.4	5.88E-07	0.00055
	Sulfate transport	0.7	6.29E-07	0.00059
	Sulfur compound transport	0.7	6.29E-07	0.00059
	Cell wall macromolecule metabolic process	2.3	9.08E-07	0.00085
	Hemicellulose metabolic process	1.9	3.21E-06	0.00303
	Xylan metabolic process	1.9	3.21E-06	0.00303
	Cell wall polysaccharide metabolic process	1.9	7.13E-06	0.00673
	Phenylpropanoid metabolic process	1.3	1.17E-05	0.01111

*Top 10 most significant processes are displayed*.

### Gene expression profiling and KEGG pathway enrichments

**Figure 6:**
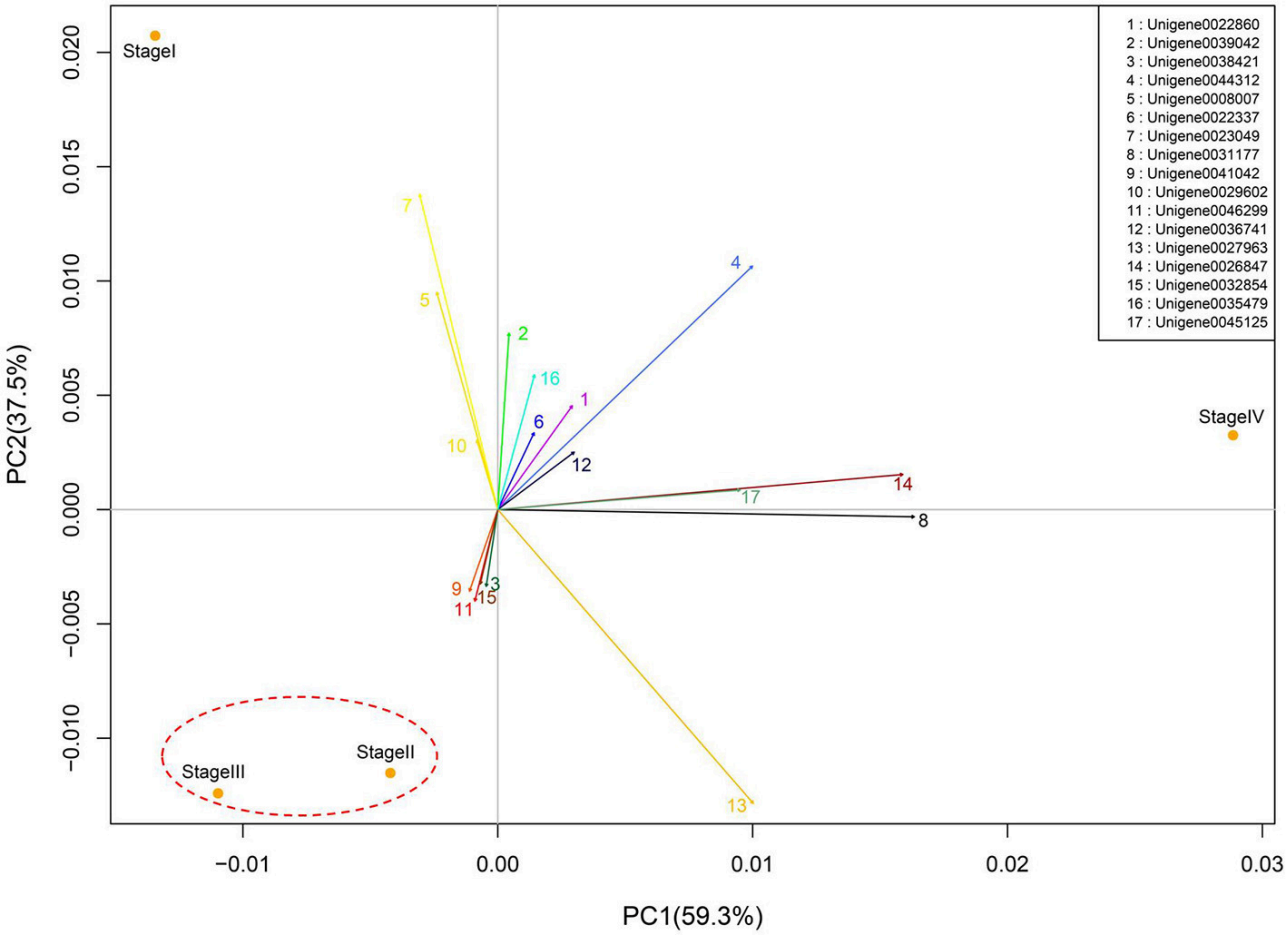
**Principal component analysis of bud stages based on the variation of global gene expression**. The numbered lines projected from the origin of coordinates indicate major genes with highest PC scores.

Gene expression patterns throughout the bud development stages were classified into 26 profiles and analysis with STEM revealed that profiles 2, 4, 5, 7, 8, 13, 20, 21, and 23 had a *P*-value lower than 0.01(Figure 6). Profile 5 included 5046 unigenes that were down-regulated during active growth in stages II and III and up-regulated during dormancy (Stage I) and growth cessation (Stage IV). The 1635 unigenes in Profile 2 had a basically similar expression pattern to those in Profile 5 but were expressed at a lowest level during rapid growth stage (Stage III). Top five most significantly enriched in Profile 5 were related to pathways like plant-pathogen interaction, hormonal signal transduction, circadian rhythm, starch, and sucrose metabolism, and linoleic acid metabolism (Table 7). Two of these pathways, hormonal signal transduction, and plant-pathogen interaction were also enriched to Profile 2 (Table 7). Hormonal signal transduction related genes fallen in Profiles 2 and 5 included the most components of the signal pathways of ABA, ethylene and cytokinin, and some components in jasmonic acid (JA) and salicylic acid (SA) signal pathways (Figure S3). In addition, ribosome, limonene and pinene degradation, stilbenoid, diarylheptanoid, and gingerol biosynthesis were among the most enriched pathways in Profile 2.

**Table 7:** **The 9 significant expression profiles and their top 5 most significantly enriched functional pathways**.

**Profile**	**Pathways**	**Unigenes involved**	***Q*-value**
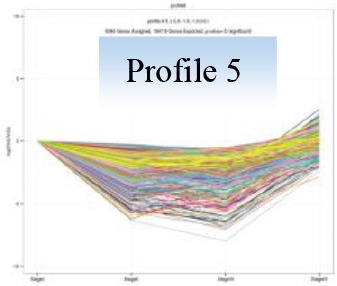	Plant-pathogen interaction	34	5.63E-6
	Starch and sucrose metabolism	34	1.52E-5
	Circadian rhythm	10	1.11E-2
	Plant hormone signal transduction	22	1.11E-2
	Linoleic acid metabolism	6	3.15E-2
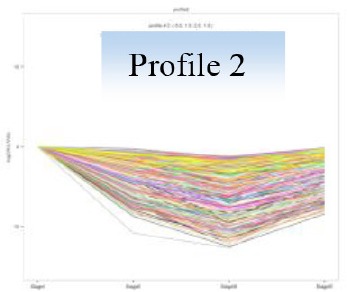	Plant hormone signal transduction	19	5.89E-6
	Ribosome	16	1.35E-3
	Plant-pathogen interaction	16	2.26E-3
	Limonene and pinene degradation	6	4.96E-2
	Stilbenoid, diarylheptanoid and gingerol biosynthesis	4	1.10E-1
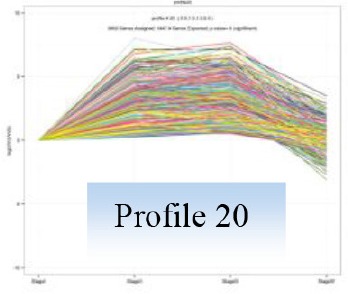	Ribosome	158	9.14E-24
	DNA replication	40	1.02E-13
	Pyrimidine metabolism	46	3.27E-4
	Purine metabolism	55	1.82E-3
	Lysine biosynthesis	10	4.03E-3
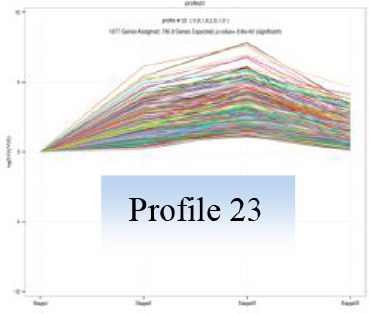	Photosynthesis	22	4.81E-22
	Photosynthesis-antenna proteins	10	1.60E-10
	Metabolic pathways	318	2.11E-6
	Porphyrin and chlorophyll metabolism	9	1.28E-4
	Starch and sucrose metabolism	37	1.42E-3
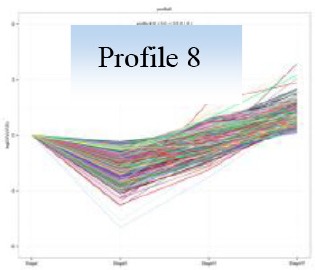	Ubiquitin mediated proteolysis	8	3.71E-2
	Metabolic pathways	39	6.31E-2
	Valine, leucine and isoleucine degradation	5	6.31E-2
	Glucosinolate biosynthesis	2	6.31E-2
	Diterpenoid biosynthesis	2	9.38E-2
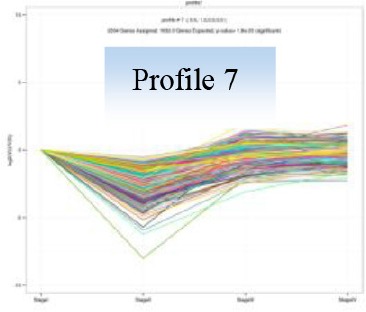	Ubiquitin mediated proteolysis	23	4.30E-5
	mRNA surveillance pathway	18	2.88E-3
	Ribosome biogenesis in eukaryotes	15	1.38E-1
	Phosphatidylinositol signaling system	8	1.56E-1
	RNA transport	19	1.56E-1
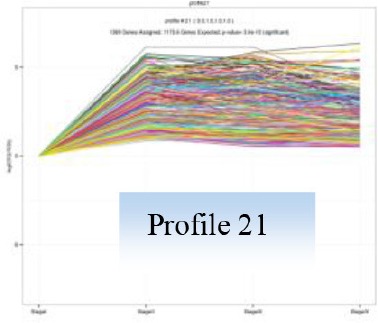	Biosynthesis of secondary metabolites	80	3.40E-11
	Metabolic pathways	117	1.48E-9
	Phenylpropanoid biosynthesis	16	1.20E-5
	Phenylalanine metabolism	11	6.90E-4
	Ubiquinone and other terpenoid-quinone biosynthesis	8	8.42E-4
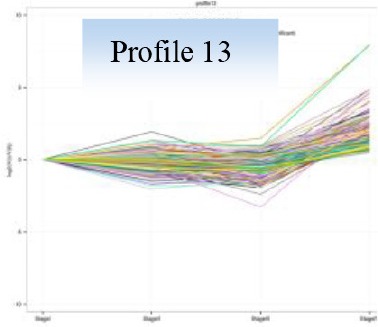	Metabolic pathway	40	3.62E-2
	Flavonoid biosynthesis	3	5.33E-2
	Fatty acid biosynthesis	4	5.34E-2
	Phenylalanine metabolism	4	1.42E-1
	Inositol phosphate metabolism	4	2.13E-1
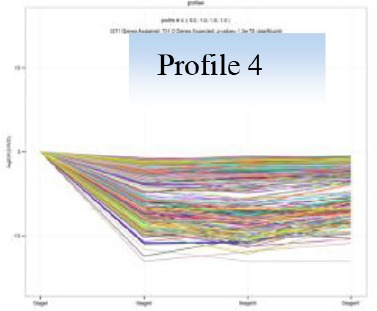	Protein processing in endoplasmic reticulum	14	0.18
	Plant hormone signal transduction	9	3.54E-1
	Spliceosome	10	3.54E-1
	Circadian rhythm	4	3.54E-1
	RNA degradation	9	3.54E-1

Profiles 20 included 3862 unigenes that were up-regulated during active growth and expressed at similar levels in Stage II and III (Figure S3). Significant pathways enriched were ribosome construction, DNA replication, pyrimidine and purine metabolism and lysine biosynthesis. These pathways are mostly related to protein synthesis and chromosome duplication that occur during cell division.

The 1077 unigenes in Profile 23 were also up-regulated during active growth stages but most highly expressed during rapid shoot elongation in Stage III. The most significantly enriched pathways included photosynthesis, antenna proteins, metabolic pathways, prophyrin and chlorophyll metabolism, starch and sucrose metabolism. The result indicated that the new growth of flush is accompanied by activation of carbohydrate metabolic pathways and construction of photosynthetic capacity. The increase in photosynthesis-related pathways agreed well with the greening of rudimentary leaves of the growing buds (Figure 2).

The 1896 unigenes in Profile 8 had the lowest expression levels during bud break but were most highly expressed during growth cessation. Pathways enriched included ubiquitin mediated proteolysis, metabolic pathway, valine, leucine, and isoleucine degradation, glucosinolate biosynthesis and diterpenoid biosynthesis. The up-regulation of ubiquitin-mediated proteolysis with growth cessation indicates massive targeted protein degradation occurred in litchi bud before it enters dormancy.

The 2034 unigenes involved in Profile 7 were lowest expressed during bud break but at similar levels among the other stages. The most significant pathways in this gene expression profile were ubiquitin mediated proteolysis, mRNA surveillance pathway, ribosome biogensis, phsphatidylinositol signaling system, and RNA transport. Hence, bud break might be accompanied by reduced ubiquitin mediated proteolysis, and generally down-regulated transcription and translation, corresponding to the largest number of down-regulated genes during the transition from Stage I to Stage II (Figure 5).

Profile 21 had 1388 unigenes that were lowest expressed during dormancy (Stage I) and similarly expressed at the other stages. The most important pathways enriched were involved in biosynthesis of secondary metabolites, metabolic pathway, phenylpropanoid biosynthesis, phenylalanine biosynthesis, and ubiquinone and other terpenpoind-quinone biosynthesis.

Profile 13 consisted of 884 unigenes that were most highly expressed during Stage IV but at similar levels at the other stages. Top enriched pathways included metabolic pathway, flavonoid biosynthesis, fatty acid biosynthsis, phenylalanine metabolism, and inositol phosphate metabolism.

The 1271 unigenes in Profile 4 were most highly expressed at dormancy and reduced to low levels during bud break and maintained the low levels at the other stages. Protein processing in endoplasmic reticulum, plant hormone signal transduction, spliceosome, circadian rhythm, and RNA degradation were the most significantly enriched pathways in this profile.

### PC analysis based on differential expressed genes

Buds at different stages could be separated by principal component (PC) analysis (Figure 6). PC1 and PC2 represented 59.3 and 37.5% of total variability of gene expression. Interestingly, PC2 separated dormant and growth stages, as dormant buds in Stage I appeared to have a positive PC2 score, while growing buds in Stage II and Stage III had negative scores. PC1, however, separated growth status, as Stage II with accelerating growth and Stage III with the fastest growth had negative PC1 scores while Stage IV with growth cessation had a positive PC1 score. Stage II and Stage III were clustered together, which agrees with their lowest number of differentially expressed genes as shown in Figure 5.

**Figure 7:**
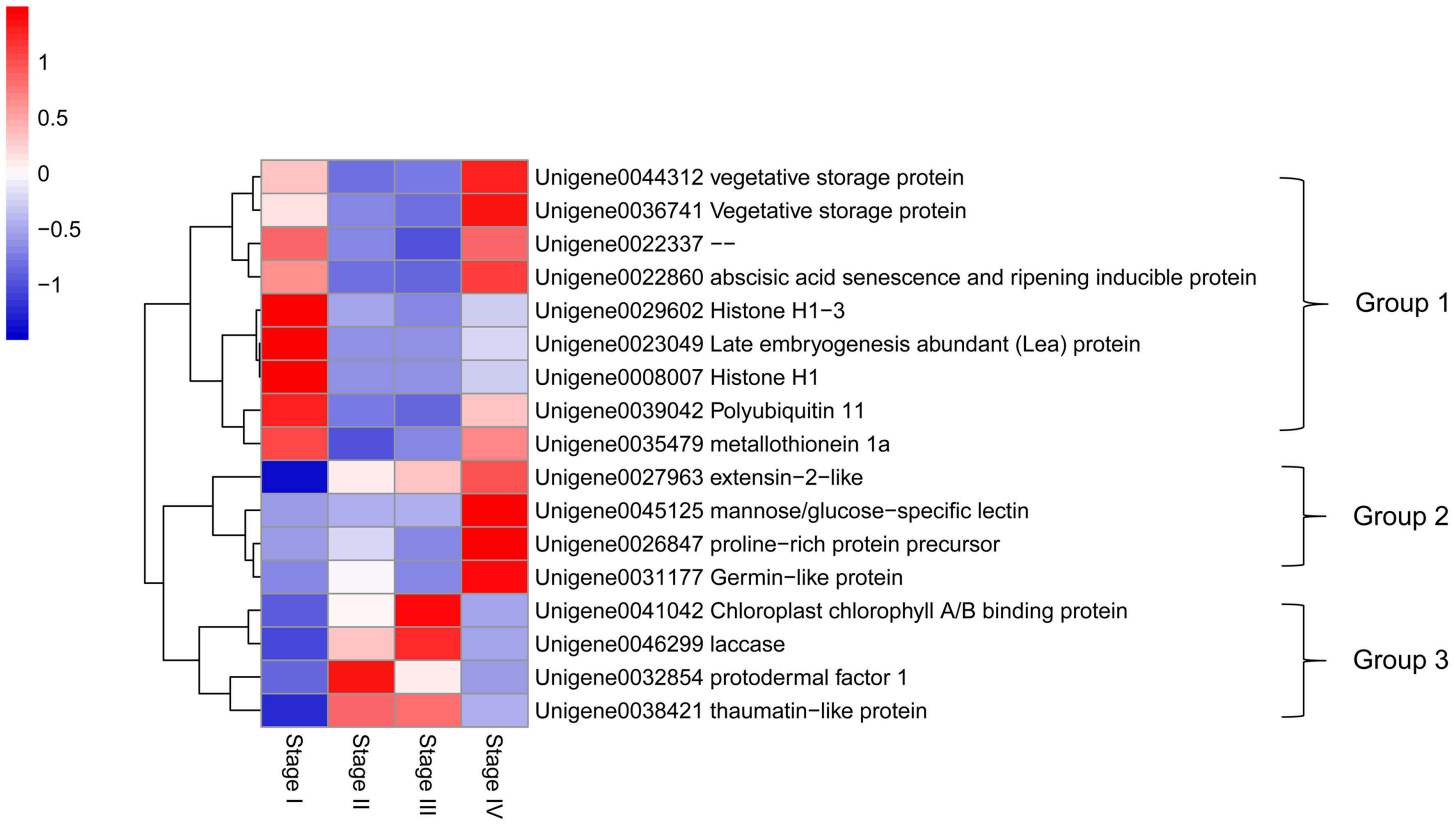
**Heat map diagram of relative expression levels and classification based on the expression patterns of the major genes with either PC1 or PC2 scores higher than 0.1**.

PC1 and PC2 scores of all unigenes are listed in Table S2. Major genes with high PC1 or PC2 scores (> = 0.1) are highlighted in Figure 6 and their expression patterns in different stages and their encoded proteins are shown Figure 7. These genes had high expression levels with RPKM values ranging from a few 100 to above 10,000 and varying drastically at different stages. Based on their expression patterns, these major genes were classified into three groups. Group 1 were genes highly expressed in dormant and growth cessation stages (stages I and IV) and down-regulated during growth (stage II and III). Proteins encoded by them included VSP (unigene00443312 and 0036741), histone H1 (unigene0008007 and 0029602), abscisic acid senescence and ripening inducible protein (ASRP, unigene0022860), late embryogenesis abundant protein (LEA, unigene0023049), polyubiquitin-like protein (PL, unigene0039042), and metallothionein 1a (unigene0035479). Group 2 included unigene0027963, 0045125, 0026847, and 0031177, which encoded extension 2-like protein, mannose/glucose-specific lectin, proline-rich protein precursor, and germin-like protein, respectively. Their expression was most active during growth cessation (Stage IV) and relatively weak in other stages. Group 3, characterized by strong expression during growth (stage II and III) and down regulation during growth cessation and dormancy, included four genes, Unigene0041042, 0046299, 0032854, and 0038421, encoding chlorophyll A/B binding protein, laccase, protodermal factor 1, and thaumatin-like protein, respectively.

### qPCR of selected genes

**Figure 8:**
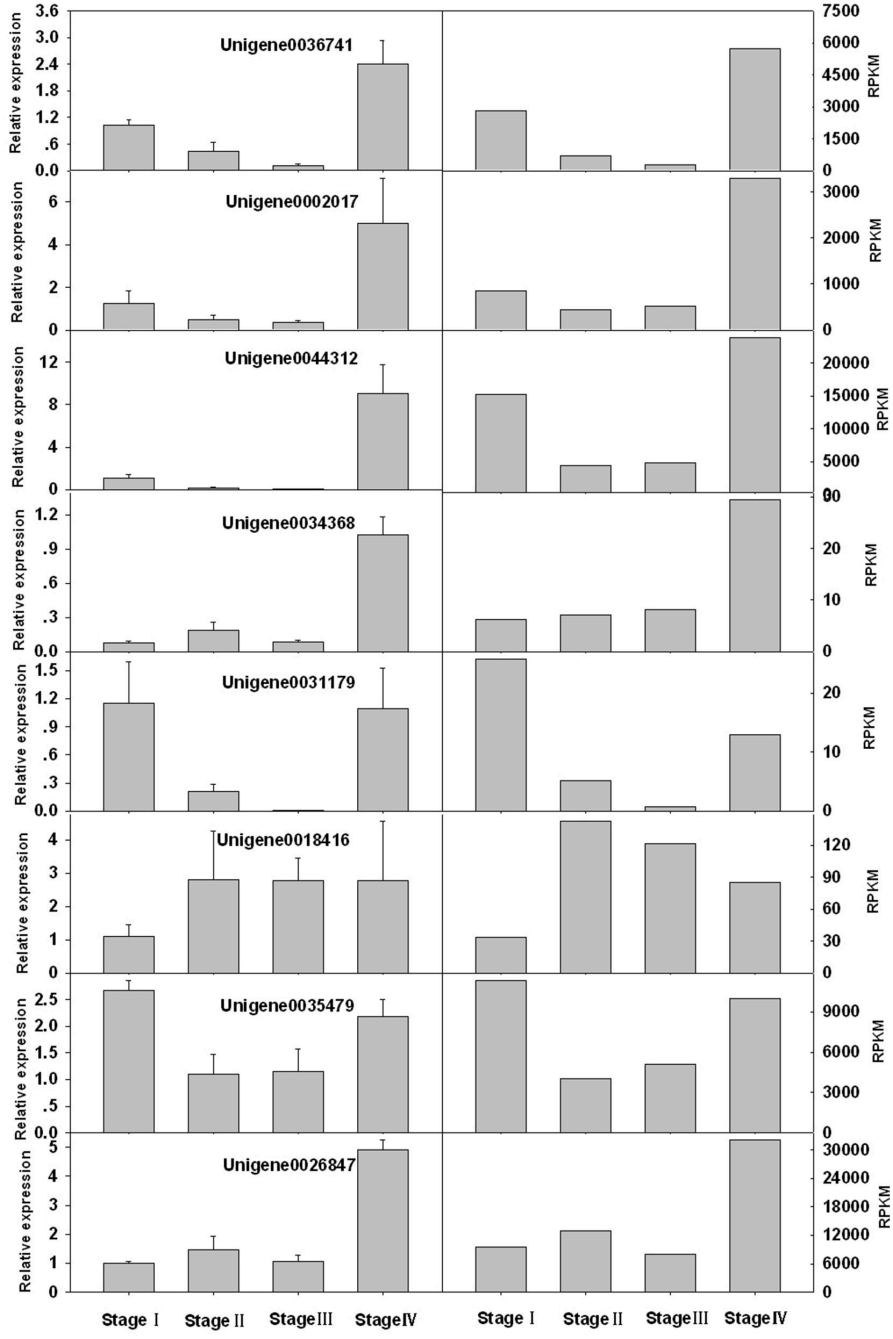
**Comparison of expression levels of 8 genes obtained by qPCR analysis (left) and by RNA-seq (RPKM values) (right)**.

**Figure 9:**
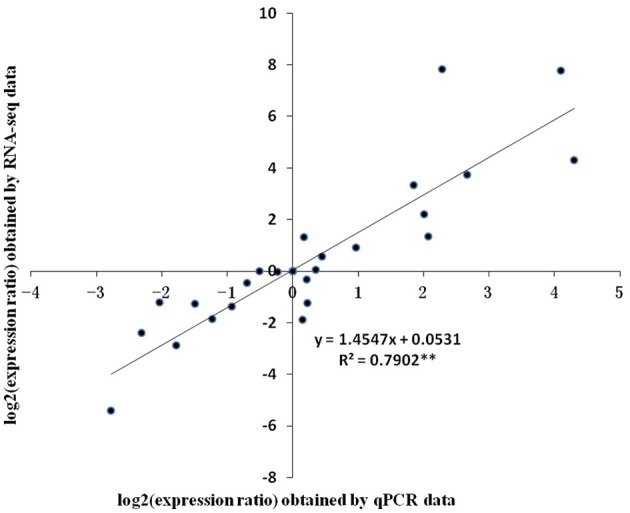
**Correlation of relative gene expression levels obtained from RNA-Seq and from qPCR**. The expression ratio was the ratio of gene expression level at one stage against the one at previous stage. Vertical bars represent standard errors of means (*n* = 3).

Eight unigenes with relatively high PC scores were selected for qPCR. Two of them, unigene0036741 and unigene0044312 were VSP, which had been reported in litchi (Tian et al., 2007). The rest unigenes were seed protein-like isoform X1 (unigene0002017 and unigene0034368), Rm1C-like cupins superfamily protein isoform 1 (unigene0031179), PRPP (unigene0026847), MT 1a (unigene 0035479) and LEA (unigene 0023049). The change patterns of the expressions of these genes at different stages obtained by qPCR generally agreed well with the RPKM values obtained by RNA-seq (Figure 8). And the relative expression levels obtained by the two methods displayed a strong linear correlation (Figure 9).

### Expression patterns of *SHORT VEGETATIVE PHASE* (*SVP*) genes

*SVP* genes are a group of MADS-Box genes that regulate dormancy, growth as well as flowering (Bielenberg et al., 2004). Three unigenes, unigene0040888, unigene0046224 and unigene0037493, annotated as *SVPs* were screened from litchi bud transcripome. They were named as *LcSVP1, LcSVP2*, and *LcSVP3*.

**Figure 10:**
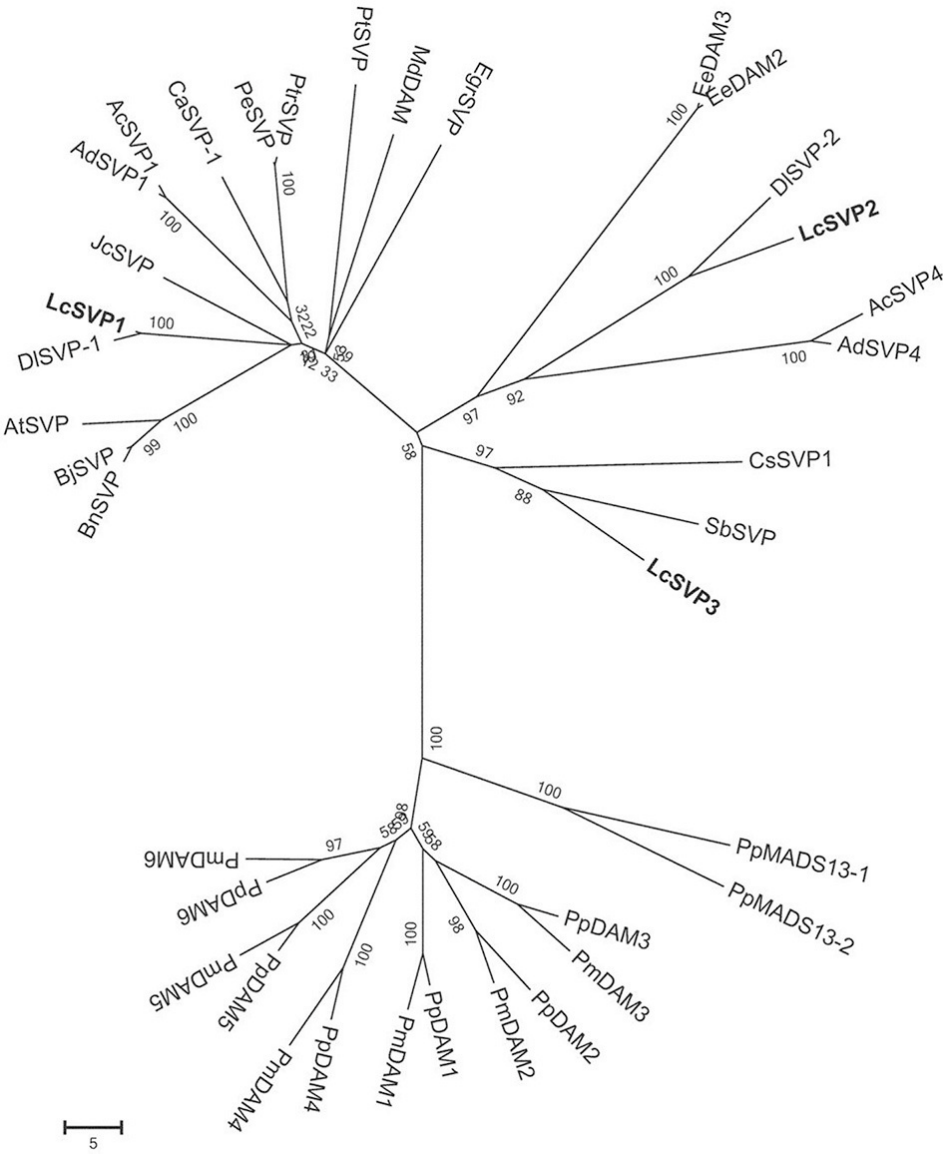
**Phylogenetic relationships between LcSVPs and SVPs of other species**. The accession numbers of these proteins in the GenBank database are as follows: *Actinidia chinensis* (AcSVP1; AFA37967; AcSVP4, AFA37970); *Actinidia deliciosa* (AdSVP1, AFA37963; AdSVP4, AFA37966); *Arabidopsis thaliana* (AtSVP, AFU85632); *Brassica juncea* (BjSVP, AFM77905); *Brassica napus* (BnSVP, AFM77907); *Coffea Arabica* (CaSVP-1, AHW58026); *Camellia sinensis* (CsSVP1, AIK35208); *Dimocarpus longan* (DlSVP-1, AIY25020; DlSVP-2, AIY25021); EeDAM2, ABY60423; *Euphorbia esula* (EeDAM3, AGB05618); *Eucalyptus grandis* (EgrSVP, AAP33087); *Jatropha curcas* (JcSVP, XP_012081656); *Malus domestica* (MdDAM, AJW82923); *Populus euphratica* (PeSVP, XP_011021843); *Prunus mume* (PmDAM1, BAK78921; PmDAM2, BAK78922; PmDAM3, BAK78923; PmDAM4, BAK78924; PmDAM5, BAK78920; PmDAM6, BAH22477); *Prunus persica* (PpDAM1, ABJ96361; PpDAM2, ABJ96363; PpDAM3, ABJ96364; PpDAM4, ABJ96358; PpDAM5, ABJ96359; PpDAM6, ABJ96360); *Pyrus pyrifolia* (PpMADS13-1, BAI48074; PpMADS13-2, BAI48075); *Citrus trifoliate* (PtSVP, ACJ09170); *Populus trichocarpa* (PtrSVP, XP_002310310); *Shorea beccariana* (SbSVP, BAN89455).

**Figure 11:**
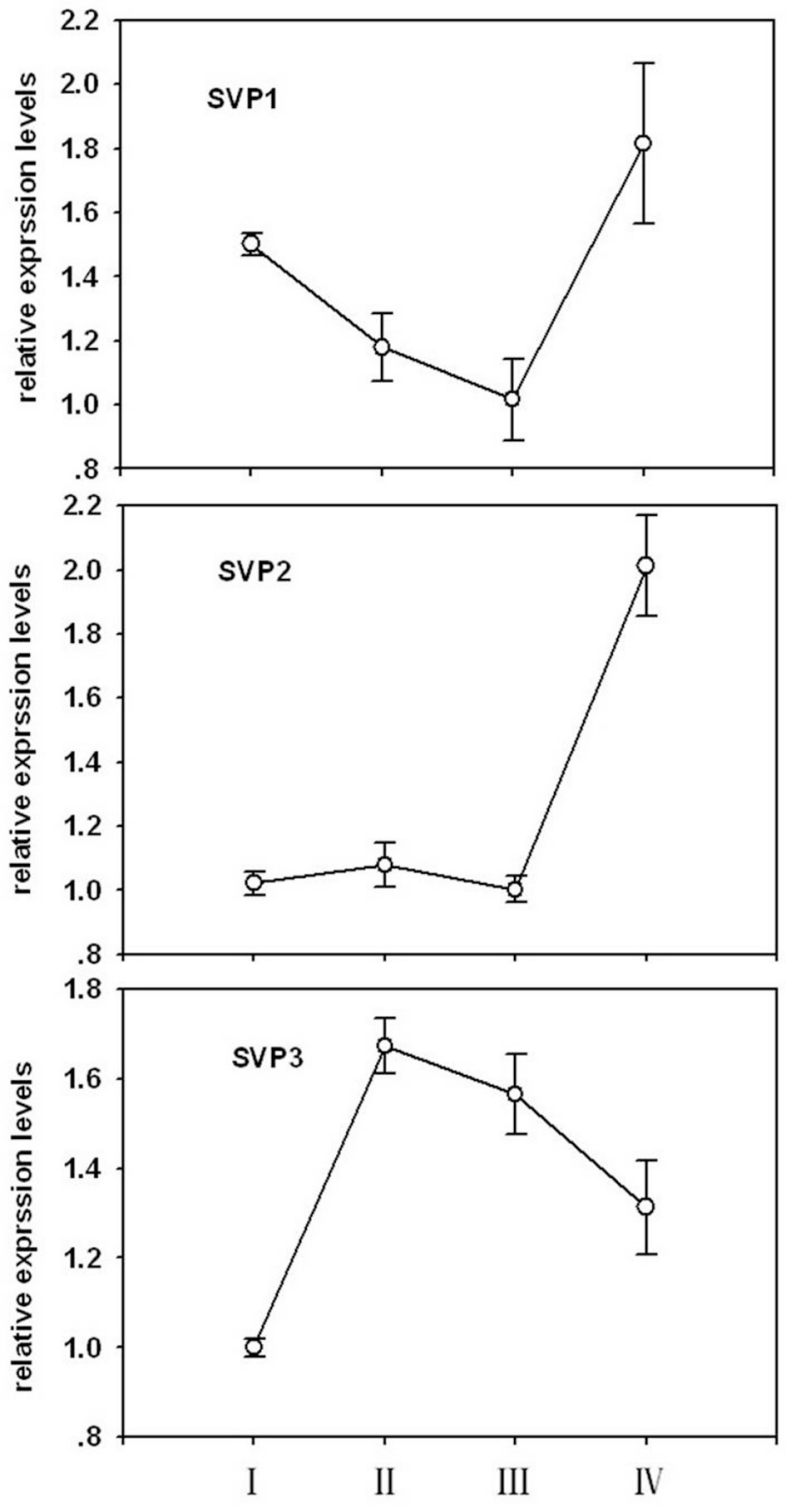
**The expression patterns of three *LcSVPs* in litchi buds**. Vertical bars represent standard errors of means (*n* = 3).

Phylogenetic relationships between LcSVPs and SVPs from other species are shown in Figure 10. The result showed that LcSVP1 and LcSVP2 had the highest homogeneity with DlSVP1 and DlSVP2 from litchi's close relative longan in the family of Sapindaceae (*Dimocarpus longan*), respectively. All DAMs from *Prunus* were clustered together and distant from the LcSVPs, which fell in different subclades. LcSVP3 was close to SbSVP from *Shorea beccariana* and CsSVP1 from *Camellia sinensis*, both of which are evergreens. LcSVP2 together with DAM3 and DAM2 from leafy spurge (*Euphorbia esula*), SPV2 from longan, SVP4 from kiwis (*Actinidia*) were clustered together. LcSVP1 together with SVP1 from longan, kiwis, and coffee, and SVPs from *Jatropha curcas, Populus trichocarpa, P. euphractica, Citrus trifoliate*, apple (*Malus domestica*), *Brassica napus, Brassica Juncea* and Arabidopsis formed a separate subclade. The expression patterns of the three *LcSVP*s were quite different (Figure 11). *LcSVP1* and *LcSVP2* were most highly expressed during growth cessation (Stage IV). However, *LcSVP3* was highly expressed during Stages II and III, but down-regulated during growth cessation.

## Discussion

Buds are important plant structures containing growing point that confers canopy growth and sexual reproduction. Although, bud development of all plants shares the same cellular processes, i.e., cell division, cell expansion and differentiation, there may be great differences in morphological, physiological, and molecular characters among species with different origins as they take different strategies to survive different environmental conditions. Extensive and intensive information has been obtained about the molecular regulation of bud development in temperate trees, whereas little is known about bud development in tropical trees. In this study, litchi was used as a model tree of tropical perennial for gene expression profiling as well as morphological characterization during bud development. With high quality sequencing and assembly data (Tables 1, 2), we were able to obtain data of global gene expression for mining the molecular processes/pathways involved in litchi bud development. Based on the knowledge of bud development in temperate trees, we were able to display the unique characters of bud development in tropical trees.

### Morphology and physiology of litchi bud at different stages

Morphologically, litchi buds have obvious differences from the “closed” buds in deciduous temperature trees whose apical meristem is well-protected by tightly imbricated scales, modified leaves called cataphylls specialized for protecting against cold temperatures in winter. The buds of litchi are naked with the apical meristem protected by rudimentary leaves instead of scales (Figure 2). As there is no need for differentiation of heat-proof scales for survival warm winter, the process of “bud set” found in temperate trees (Anderson et al., 2005) is absent in litchi. Bud growth in temperate deciduous trees is based on annual cycles, while the recurrent bud growth of litchi has shorter but more frequent cycles (Fu et al., 2014), alternating between rest or developmental dormancy and growing states even in constant growth-favorable conditions (Hieke et al., 2002). Therefore, the developmental dormancy of litchi bud is different from the winter endodormancy, which is induced by environmental cues such as short-day photoperiod or low temperatures (Davis, 2002; Arora et al., 2003; Anderson et al., 2010). With the release of dormancy, the breaking buds of litchi displayed unique morphological changes. The rudimentary leaves swelled, turned green, and gradually opened exposing the inner leaf primordia (Figure 2). Unlike the bud scales that are discarded after bud break in deciduous trees, the rudimentary leaves in litchi continued to develop into pinnate leaves after bud break. The outgrowth of several new leaves occurred almost at the same time with the rapid elongation of the new flush that continued until leaflet expansion and maturation (Fu et al., 2014). As young leaves expanded, bud growth/flush elongation ceased and the later-coming underdeveloped leaves in upper positions were aborted (abscised; Figure 2H), indicating strong competitions between bud growth and leaf growth and among growing leaves. Then, the tip rudimentary leaves or leaf primodia shrank, turned brown, and looked dry (Figure 2I), probably due to desiccation. The bud reentered dormant status. Hence, litchi bud dormancy is preceded by growth cessation, which is in some way similar to the bud set process in temperate deciduous trees. Release of bud dormancy in litchi occurs spontaneously when the young leaves of the new flush are fully matured (Fu et al., 2014), which is quite different from the dormancy release in temperate deciduous trees in response to environmental cues such as chilling (Arora et al., 2003; Anderson et al., 2010). In addition, bud dormancy release in litchi is not associated with flower development as commonly found in temperate deciduous trees, although it renders competence to chilling temperatures (Batten and McConchie, 1995). Like litchi, the evergreen citrus trees also undergo cyclic flush growth, but with flush growth cessation, the terminal buds of citrus abscise, a process called “self-pruning” (Zhang J. Z. et al., 2014), which is rarely observed in litchi.

Without cost for growth, it is understandable that dormant buds of litchi had the lowest respiration rate (Figure 3). Interestingly, breaking buds of litchi had the highest respiration rate with an oxygen consumption rate even higher than that in buds of active growth (Stage III). The result suggests that initiation of bud growth requires more energy than growth itself in litchi. The result reveals fundamental difference in physiology between bud break in litchi and in some temperate species such as grape, where a period of hypoxia or respiration stress that induces anaerobic respiration is required to initiate bud growth from dormant status (Halaly et al., 2008; Vergara et al., 2012). This requirement might be related to bud structure of grape, which is closed by the scales with poor oxygen delivery to the meristem, and the anaerobic respiration enables energy supply under low oxygen availability before bud scales are broken by the emerging new growth. In contrast, the naked buds of litchi have no need for anaerobic respiration, and bud break commences with increased oxygen consumption (Figure 3).

### Global transcriptomic changes during bud development

PC analysis based on global gene expression distinguished buds at different stages in grape (Díaz-Riquelme et al., 2012). In litchi, least difference in global gene expression was found between breaking buds at Stage II and growing buds at Stage III, which were most closely clustered together (Figure 6), whereas dormant buds at Stage I and growth cessation buds at Stage IV were clustered far from each other as well as from buds at Stage II and Stage III, suggesting great differences in global gene expression and thus functions among buds in those stages. The result agreed with the stage-to-stage comparison of differentially expressed genes (Figure 5). Our result showed that more differentially expressed unigenes were up-regulated during dormant phase than actively growing phase in litchi bud (Figure 5), indicating high transcriptional activity in dormant buds despite their low metabolic activity.

We separately carried out functional GO biological process enrichment analyses for up-regulated and down-regulated gene groups during transitions between bud developmental stages (Tables 3–6). The results indicated that litchi bud development, especially dormancy entrance and removal might be subject to epigenetic regulation that involves chromatin (histone lysine) methylation. Increased activities of macromolecule methylation, esp. cytidine methylation during bud dormancy release induced by 6-benzylaminopurine were reported in tobacco (Schaeffer and Sharpe, 1970). Similarly, Santamaría et al. (2011) carried out comparative transcriptome analysis of dormant and non-dormant chestnut (*Castanea sativa*) buds and found bud dormancy might be under epigenetic control. However, their study focused on roles of histone ubiquitination, acetylation and phosphorylation, instead of methylation. Increased attention has been paid upon epigenetic regulation of bud dormancy in perennials, especially on key regulator genes of dormancy such as DAM (Ríos et al., 2013). Chromatin modification of DAMs has been reported in peach and leafy spurge, where trimethylation at H3K27 and decreased trimethylation at H3K4 of these genes led to expression reduction and dormancy release (Horvath et al., 2010; Leida et al., 2012). In viewing the large evidence of chromatin remodeling involved in dormancy control, Considine and Considine (2016) suggested that the depth of dormancy might be governed by the order of heterochromatin state. Further studies are needed to clarify the details about epigenetic control of bud dormancy in litchi.

The results also showed that salicylic acid and programmed cell death might be involved in regulation of bud development cycle in litchi, as bud dormancy release occurred with the down-regulation of the response to salicylic acid and programmed cell death (Table 3), while growth cessation took place with the up-regulation of the two processes (Table 5). Occurrence of programmed cell death has also been reported in citrus bud during the process of self-pruning (Zhang J. Z. et al., 2014). The role of salicylic acid in bud development control awaits detailed study.

Analysis with STEM displayed 9 significant gene expression profiles during bud development. Unigenes that were up-regulated during dormant or growth cessation stages or down-regulated after bud break (Profile 5 and 2 in Table 7), were significantly enriched into hormonal signal transduction, circadian rhythm, plant-pathogen interaction. The results indicate that bud growth cessation and dormancy entrance involve significant changes in hormonal signaling (Figure S4). It is understandable that major components in signal pathways of ABA and ethylene were up-regulated during growth cessation and dormancy, as these hormones are the key players in inducing growth cessation and dormancy entrance in deciduous trees (Ruttink et al., 2007; Horvath et al., 2008; Olsen, 2010). The two hormones are also involved in organ abscission (Gomez-Cadenas et al., 1996; Zhu et al., 2011), which agrees with the abscission of rudimentary leaves observed during growth cessation in litchi (Figure 2H). JA and SA serve as defense hormones that mediate the defense responses to insect and pathogen attacks (Smith et al., 2009). The up-regulation of signal pathways of the two hormones together with the activation of genes involved in plant-pathogen interactions (Table 7) and stress responses may contribute litchi buds resistance to stresses, especially to pests and disease as they enter dormancy status. Indeed, new growth of flush in litchi is more vulnerable to pest and pathogen attacks than dormant buds, and therefore, occurrence of new flush is a crucial period for pest and disease control. In this sense, dormancy confers increased stress resistance to litchi bud and is similar to the dormancy in temperate trees, which is developed for coping with the stress conditions in winter (Rohde and Bhalerao, 2007). In litchi, the alternation between stress-susceptible growth and stress-resistant dormant states as well as relatively synchronized outgrowth of several leaves in one flush cycle avoids the risk of constant pest and pathogen attacks and might be an important strategy of litchi for survival in a tropical environment, where stresses of pests and pathogens are ever present.

Strangely, the major components of cytokinin signal pathway were down-regulated during bud break and active growth stages (Figure S4), when cell division was active. The result seems contradictory to the well-accepted functions of cytokinins, which induce cell division by promoting G2 to M transition (Skylar and Wu, 2011). O'Hare and Turnbull (2004) found pre-bud break accumulation of a root-delivered cytokinin (zeatin riboside) in dormant litchi bud and suggested bud break occurred when endogenous cytokinin level reached a critical level. However, they found cytokinin level decreased sharply after bud break. Continuous accumulation of cytokinin in litchi buds was only found during floral differentiation after a period of exposure to chilling temperatures (Chen, 1991). Application of exogenous cytokinin induced bud break but fail to induce flush emergence (O'Hare and Turnbull, 2004). Our preliminary results showed that application of exogenous cytokinins (zeatin and N(2-chloro-4-pyridyl)-N′-phenylurea, CPPU) during flush emergence suppressed flush elongation and leaf expansion (unpublished results). Hence, down-regulation of cytokinin signal pathway after bud break might be a prerequisite for the up-coming flush growth. Detailed roles played by cytokinins at different phases of bud development need further study.

The induction of growth cessation and dormancy by short photoperiod (Arora et al., 2003; Anderson et al., 2010) suggests that the development of bud dormancy is subject to circadian control in temperate deciduous trees. There has been no report about the photoperiod dependence of growth cycle in litchi. Interestingly, our result showed the pathway of circadian rhythm was up-regulated during bud dormancy and growth cessation (Table 7), indicating that some processes in dormant buds might be subject to circadian control. Our previous work showed that continuous shading treatment during bud dormancy strongly suppressed bud break of litchi (Mo et al., 2013; Zhou et al., 2014), suggesting dormant bud is able to respond to light for dormancy removal. It is worthy to take an insight study of circadian control of litchi bud development.

Ubiquitin-mediated proteolysis, a mechanism for targeted protein degradation and an essential pathway for protein turnover in eukaryotic cells (Ciechanover et al., 2000; Vierstra, 2003), was highly expressed during growth cessation (Table 7), suggesting transition to bud dormancy involves massive selective protein degradation and protein turnover. Ubiquitin-mediated proteolysis controls a wide range of cell processes in plants (Vierstra, 2003). For example, it participates in the termination of mitosis and cell differentiation (Marrocco et al., 2010). Therefore, the transition to bud dormancy, which involves suspension of cell division, agrees with the up-regulated ubiquitin-mediated proteolysis observed during this period.

Profile 20 includes unigenes that were up-regulated during bud break and growth (Stage IV). They were significantly enriched to pathways related to DNA and protein synthesis, such as ribosome, DNA replication, purine and pyrimidine metabolism, and homologous recombination, which are all involved in cell division. The results are consistent with the active cell division occurring during bud break and growth in Stage II and III.

Unigenes in profile 23, which were also activated after bud break but most actively expressed at Stage III. Pathways significantly enriched included photosynthesis, antenna protein, porphyrin and chlorophyll metabolism, carbohydrate metabolism. Porphyrin, and chlorophyll metabolism is related to biosynthesis of chlorophylls. Hence, buds after break in litchi are characterized by build-up of photosynthetic mechanisms. The activated pathways related to photosynthesis agreed with the greening of bud from bud break (Figure 2). With the output of photosynthates, starch, and sucrose metabolism were also activated in buds after break, which was consistent with the increased respiration rate (Figure 3), so that the increased energy and material demand for growth could be satisfied.

### Expression pattern of major genes with different functions

PC analysis highlighted 17 major genes with high PC scores (> = 0.1; Figure 6). Nine of them, highly expressed during dormancy and growth cessation, encode proteins which are accumulated in large quantities in plants as nutrition reserves (VSP and LEA), as structural components in chromosomes (histone H1) or for protection against stresses (ASRP, LEA and MT) and for protein turn-over (polyubiquitin). Four genes, specifically highly expressed in growth cessation stage, encode an extension-like protein, a mannose/glucose-specific lectin, a proline-rich protein and a germin-like protein, which are all located in cell walls and play important roles in wall construction, defense or cell extension control (Wilson and Fry, 1986; Bradley et al., 1992; Bernier and Berna, 2001; Mann et al., 2001). Four of the 17 genes were highly expressed during growth. One of them encodes a chlorophyll binding protein. It's up-regulation during growth agrees with the greening of bud after bud break. A second gene encodes laccase, a copper-containing enzyme catalyzing oxidation and polymerization of phenols as found in formation of lignin (Dean and Eriksson, 1994). A third gene encodes protodermal factor 1, which has been shown to play a crucial role in the initiation and growth of trichomes in cotton (Deng et al., 2012). The forth one encodes a thaumatin-like protein, a type of pathogenesis-related protein involved in defense (Ruiz-Medrano et al., 1992). Hence, the 4 major genes up-regulated during growth had diversified functions. Obviously, a majority of the 17 major genes are related to defense or stress resistance and most of the defense genes are actively expressed during growth cessation and dormancy. The results suggest that growth cessation and dormancy might be an important developmental period for building up proteins for nutrition reserve and defense. This agrees with what is revealed from KEGG pathway enrichment analysis of gene expression profiles (Table 7).

### Expression patterns of MADS-Box containing *SVP* genes

Bud growth, dormancy, and flowering in perennials are controlled by a group of genes called DAMs (Bielenberg et al., 2004; Mazzitelli et al., 2007; Horvath et al., 2010; Ubi et al., 2010; Sasaki et al., 2011; Falavigna et al., 2014), which are homologous to the SVPs that regulate flowering in annuals, such as Arabidopsis (Lee et al., 2007). Studies have shown that different members of *DAMs*/*SVP*s have distinct functions. In peach, 6 members of PpDAMs (PpDAM1–PpDAM6) have been identified, among which PpDAM1, PpDAM2, and PpDAM4 are associated with seasonal growth cessation and bud set (Li et al., 2009), while PpDAM5 and PpDAM5 are involved in maintenance of endodormancy (Yamane et al., 2011a) and suppression of flowering (Yamane et al., 2011b). In kiwifruit (*Actinidia spp*.), four *AcSVP* members showed distinct seasonal and spatial patterns of expression, although they were all down-regulated during bud break and flower differentiation, and only AdSVP2 and AdSVP3 were shown to rescue the Arabidopsis *svp* mutant that displayed early flowering (Wu et al., 2012). In this study, three SVPs were identified in litchi buds. They are clustered in different subclades (Figure 10) and relatively distant from the DAMs from *Prunus*, which formed a separate subclade. *LcSVP2* was highly expressed during growth cessation stage (Figure 11), suggesting that it is involved in entrance to dormancy in litchi bud. *LcSVP1* was highly expressed during dormancy and growth cessation and was down-regulated during bud growth, indicating this gene might be responsible for growth cessation and dormancy maintenance. The expression of *LcSVP3* was low during growth cessation and dormancy stages, which was opposite to that of *LcSVP1*. The results indicate distinct functions of *LcSVP3*, which awaits further clarification.

## Conclusions

Bud development of tropical litchi undergoes morphological changes distinct from that of deciduous trees and that of evergreen citrus. In-depth RNA sequencing revealed global changes in gene expression during bud development in litchi, which highlighted significant changes in pathways of hormone signaling, circadian rhythm, disease resistance, photosynthesis, cell division and carbohydrate metabolism during phase transitions of bud development. The results suggest bud development, especially developmental dormancy removal and reentry, might be subject to epigenetic control which involves chromatin methylation, and that programmed cell death participate growth cessation and dormancy entry in litchi bud. The results also indicate that bud dormancy development is accompanied with up-regulation of defensive pathways and therefore the alternation between growth and dormancy provides tropical trees a survival strategy to deal with the ever-existing threat of pests and diseases in tropical climate. The oxidative stress or anaerobic respiration observed in deciduous trees during dormancy release was not found in litchi buds. Three SVP regulator genes, reportedly controlling dormancy and flowering in other plants, were cloned from litchi bud. The expression of *LcSVP1* and *LcSVP2* correlated well with growth cessation and dormancy maintenance, while *LcSVP3* seemed related to bud break and growth. Genome-wide transcriptomic analysis in this study opened new research scopes for the study of the molecular regulations of bud development and growth rhythm in tropical perennials.

## Author contributions

HZ, HL, and BL have participated the field experiments, gene expression analyses, data processing and analysis, draft writing. HX contributed RNA sequencing, *de novo* assembly, and bioinformatic analyses. HW, XH contributed the designing of the experiments, refined data analysis, and critical review and refining of the manuscript.

### Conflict of interest statement

The authors declare that the research was conducted in the absence of any commercial or financial relationships that could be construed as a potential conflict of interest.

## Supplementary material

Figure S1 **Procedures of sample process for RNA sequencing (A), *de novo* assembly (B), unigene annotation and global gene expression analyses (C)**.

Figure S2 **GO (A) and COG (B) functional classification of all unigenes**.

Figure S3 **The 26 gene expression profiles subject to STEM analysis**. Colored profiles are significant with *P* < 0.01.

Figure S4 **Hormone signal pathways in litchi buds**. Components in red frames were up-regulated *during* growth cessation and dormancy (Profiles 5 and 2 in Table 7).

Table S1 **Unigenes and their primer pairs for real-time PCR**.

Table S2 **PC1 and PC2 scores of all unigenes**.

## Abbreviations

ABA: abscisic acid
ASRP: abscisic stress ripening protein
HC: hydrogen cyanamide
ATP: adenine triphosphate
cDNA: complementary deoxyribonucleic acid
COG: Clusters of Orthologous Groups of proteins
CT: cycle threshold
DAM: dormancy associated MADS-box
GA: gibberellins
GL: germin-like protein
GO: Gene Ontology
HSP: heat shock proteins
JA: jasmonic acid
KEGG: Kyoto encyclopedia of Genes and Genomes
LEA: late embyogenesis abundant proteins
NCBI: The National Center for Biotechnology Information
PC: principal componment
PCA: principal component analysis
PCR: polymerase chain reaction
PL: polyubiquitin-like protein
PRPP: proline-rich protein precursor
RMA: Robust Multiarray Analysis
RNA: ribonucleic acid
RNA-seq: ribonucleic acid sequencing
RPKM: reads per kilobase of exon model per million mapped reads
SA: salicylic acid
STEM: Short Time-series Expression Miner
SVP: short vegetative phase
VSP: vegetative storage protein
WEGO: Web Gene Ontology.
