# Supplementary figures and images for: Morphological Characterization and Gene Expression Profiling during Bud Development in a Tropical Perennial, Litchi chinensis Sonn
Source: Front Plant Sci. 2016 Oct 26;7:1517. doi: 10.3389/fpls.2016.01517 (PMC5080376; doi:10.3389/fpls.2016.01517)

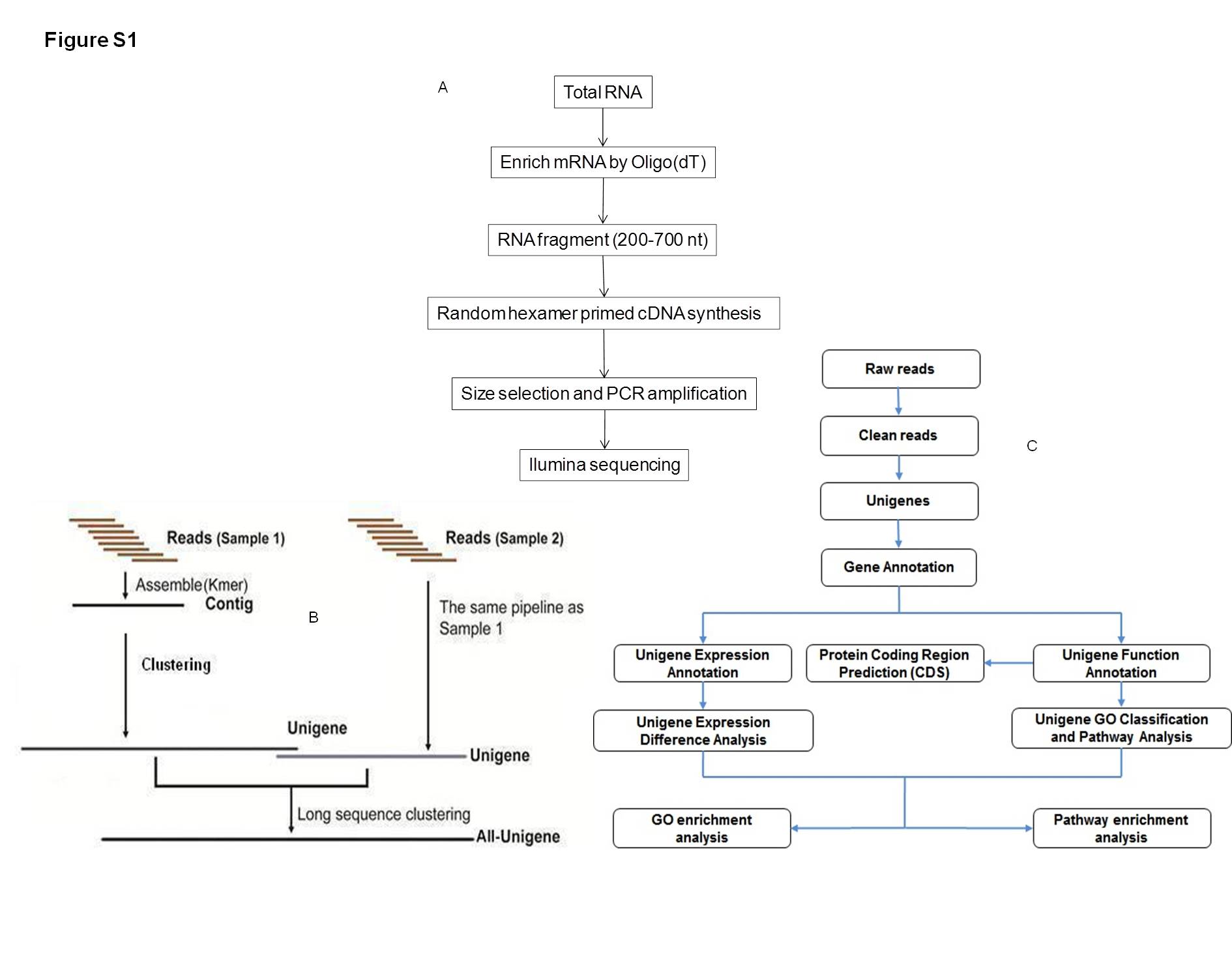

Supplement: Figure S1 — Procedures of sample process for RNA sequencing (A), de novo assembly (B), unigene annotation and global gene expression analyses (C). [file Image1.JPEG]

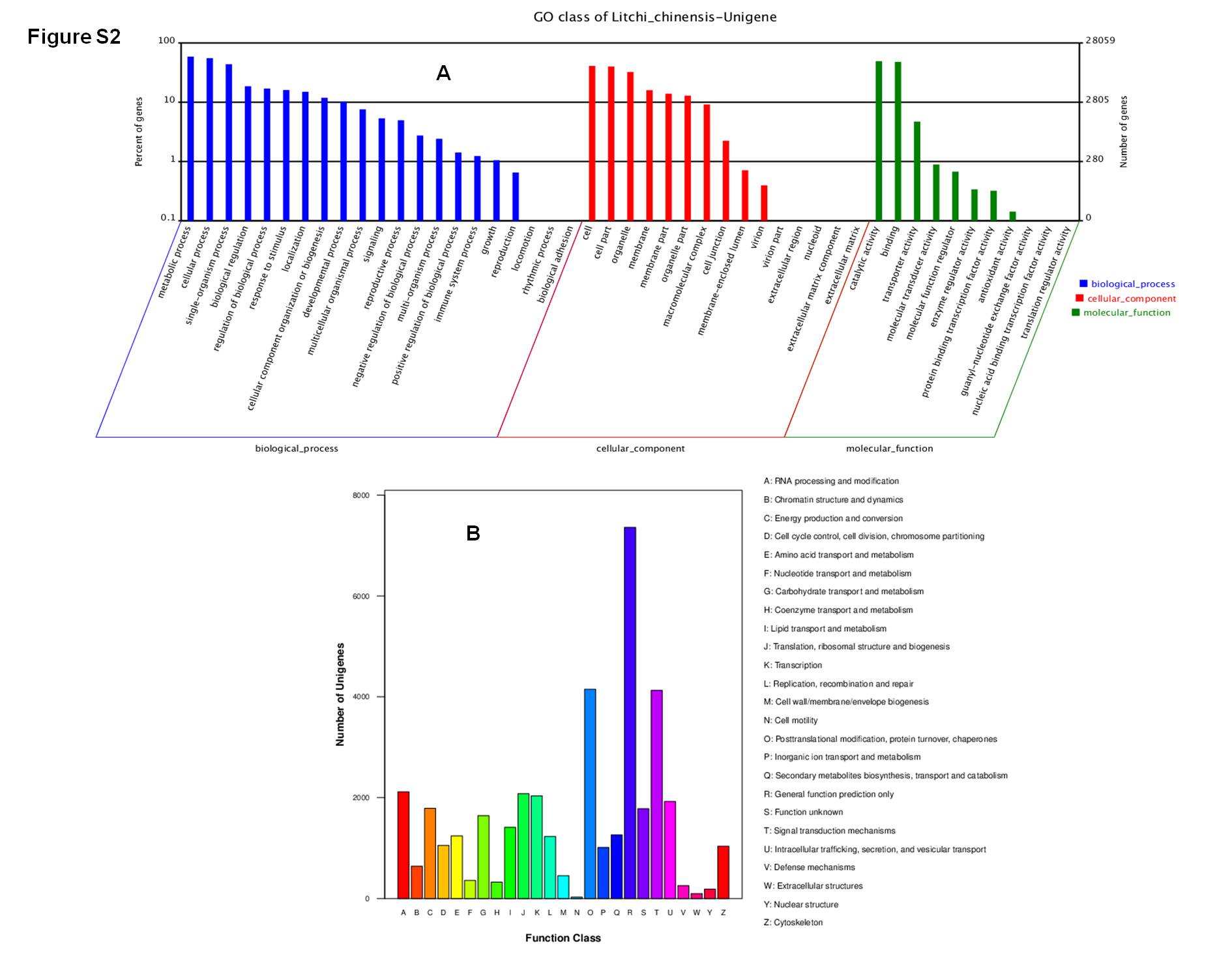

Supplement: Figure S2 — GO (A) and COG (B) functional classification of all unigenes. [file Image2.JPEG]

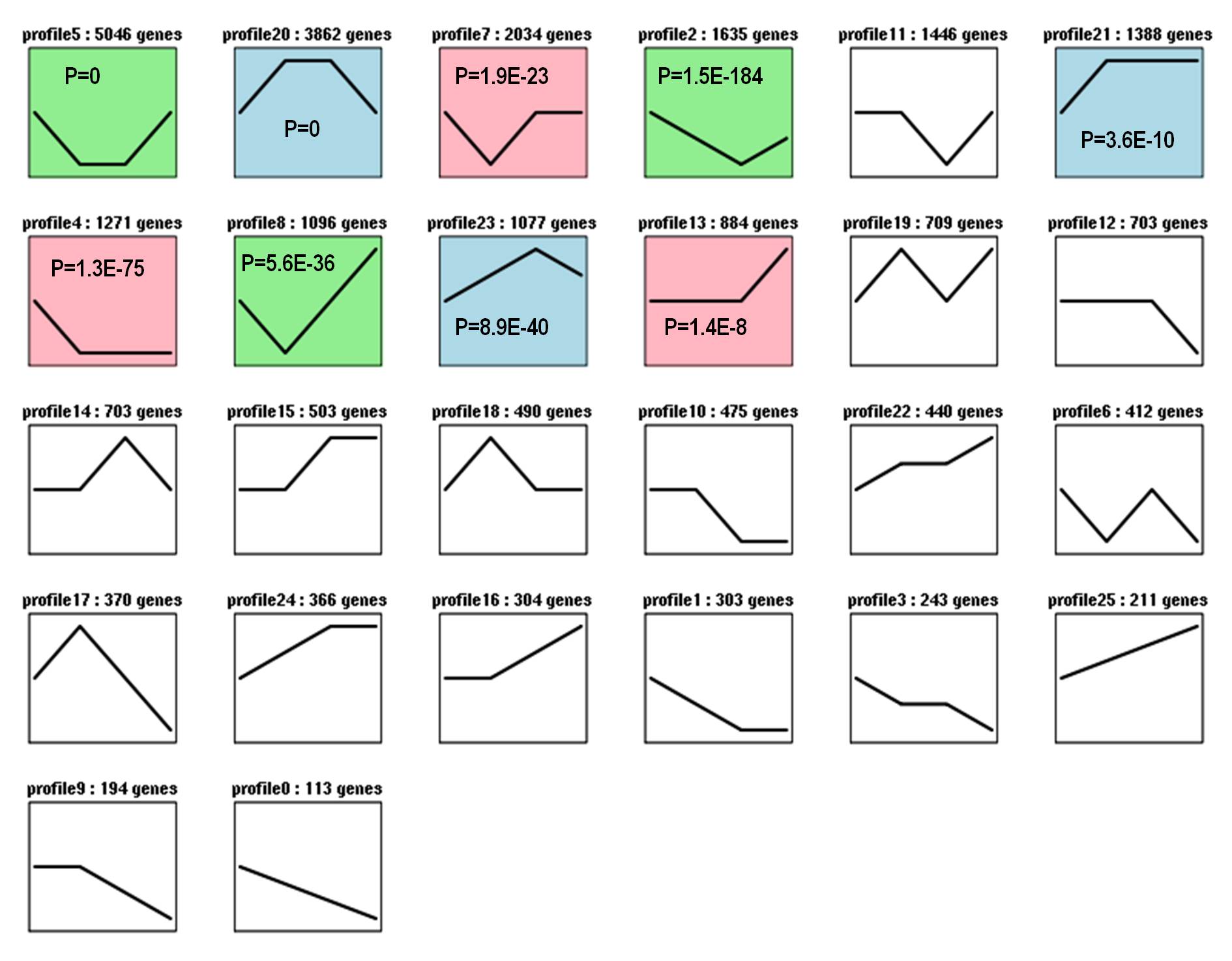

Supplement: Figure S3 — The 26 gene expression profiles subject to STEM analysis. Colored profiles are significant with P < 0.01. [file Image3.JPEG]

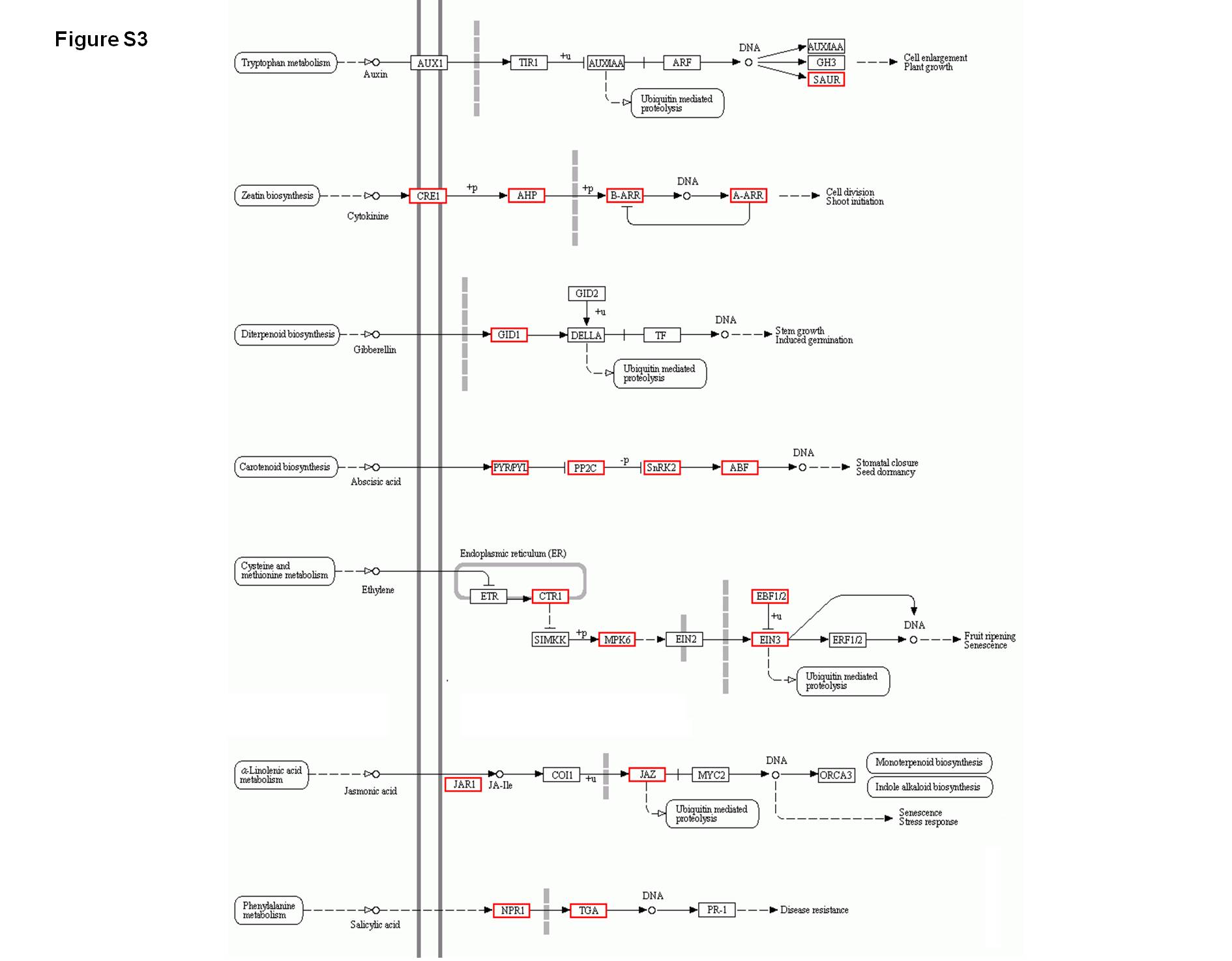

Supplement: Figure S4 — Hormone signal pathways in litchi buds. Components in red frames were up-regulated during growth cessation and dormancy (Profiles 5 and 2 in Table 7). [file Image4.JPEG]
