# Supplementary material for: Morphological Characterization and Gene Expression Profiling during Bud Development in a Tropical Perennial, Litchi chinensis Sonn
Source: Front Plant Sci. 2016 Oct 26;7:1517. doi: 10.3389/fpls.2016.01517 (PMC5080376; doi:10.3389/fpls.2016.01517)
Supplement: Table S1 — Unigenes and their primer pairs for real-time PCR. [file Table1.DOCX]

| Gene ID | Protein encoded | F（5'→3'）sequence | R（3'→5'）sequence |
| --- | --- | --- | --- |
| Unigene0036741 | VSP1 | TCATTTCTCCCAAACCCATT | CCTCACTTTCCACGACAACAG |
| Unigene0002017 | SPL X1-1 | TTTGCGTGTCCAAGTGAAGTT | CGAAGTTGGCGTAGTGGTAA |
| Unigene0044321 | VSP3 | ATGACATCAATGGCGACGAAG | TGACAACAGGCACGGAGGT |
| Unigene0034368 | SPL X1-2 | ACGGCGATAATGTAGGACG | GAGCAGTAGGGAGACAGATTTG |
| Unigene0031179 | GL1 | TGTGGCTTGCTTTGGGTC | TTTACTCCTTCGGTTTCGG |
| Unigene0026847 | PRPP | AAATCAGCCAGCCCTTG | CATCCCACCGTCCTTGGT |
| Unigene0035479 | MT | TCAGTGGTTGTGGTGGTC | TTGTTTCTTTGGCTGAAGTG |
| Unigene0023049 | LEA | ACCCTCCCCTACCCGATAC | TGGTTCCTGCCACCCTAC |
| Unigene0040888 | SVP1 | CTTTCTGTTCTTTGCGATGC | TTCTCAAGGTTCTTGGAGTGC |
| Unigene0046224 | SVP2 | AGGAAATGCTAACCCCAACT | AGACAAGGGAACAACAACTGAT |
| Unigene0037493 | SVP3 | CCGACTCCATGAAGACGAC | TGATTGAGACTAAGGGTGAACG |
|  | Lcactin | AGTTTGGTTGATGTGGGAGAC | TGGCTGAACCCGAGATGAT |

**Table S1 Unigenes and their primer pairs for real-time PCR**
